# Genetic diversification of Pseudomonas fluorescens maintained by multi-niche selection within biofilms

**DOI:** 10.1128/aem.02499-25

**Published:** 2026-03-30

**Authors:** Abigail M. Matela, Colton W. Siatkowski, Changhua Yan, Sachin Thiagarajan, Erin M. Nawrocki, Vaughn S. Cooper

**Affiliations:** 1 University of Pittsburgh School of Medicine, Pittsburgh, Pennsylvania, USA; 2 Department of Microbiology and Molecular Genetics, Center for Evolutionary Biology and Medicine, University of Pittsburgh, Pittsburgh, Pennsylvania, USA

**Keywords:** biofilms, science education, evolution, genomics, cyclic-di-GMP, niche differentiation

## Abstract

We established a research-education partnership, EvolvingSTEM, that currently provides thousands of secondary school students the opportunity to conduct authentic research experiments centered on microbial evolution each year. Providing high school students access to research experiences improves learning, can have positive and long-lasting impacts on their attitudes toward science, and gives them the opportunity to make impactful scientific contributions. Through EvolvingSTEM, students evolve populations of *Pseudomonas fluorescens* in a bead model that includes daily cycles of bacterial dispersal, attachment, and biofilm growth, and observe heritable changes in colony morphology. We sequenced the genomes of 69 evolved clones with unique colony morphologies and identified parallel mutations in genes known to regulate or affect biofilm growth (*wsp*, *yfiBNR*, *morA*, and *fuzY*). We also uncovered novel adaptations: loss-of-function mutations in phosphodiesterase PFLU0185 that did not alter colony morphology and mutations affecting periplasmic disulfide bond formation producing small colonies. PFLU0185 mutants rapidly and consistently reached high frequencies, and phenotyping revealed roles in cyclic di-GMP regulation, biofilm formation, and motility, prompting us to name this gene *bmo* (biofilm and motility regulator). Competition experiments and microscopy demonstrated that *bmo* mutants are ecological generalists that compete with their ancestor and specialist mutants. Consequently, phenotypic diversity is maintained, with smooth (ancestral and *bmo*) colonies consistently outnumbering wrinkly and fuzzy variants. This study advances our understanding of biofilm genetic architecture while demonstrating that student-led research can uncover mechanisms of microbial adaptation relevant to *Pseudomonas* infection biology.

## IMPORTANCE

Bacterial biofilms dominate microbial life; however, their evolutionary genetics remain incompletely understood. Extensive replication of biofilm selection experiments by secondary school students can provide valuable insights into mechanisms of adaptation. Notably, mutations in a conserved phosphodiesterase, PFLU0185/*bmo*, dominate evolved populations without changing colony morphology. Phenotypic assays reveal that *bmo* plays a generalist role and competes with its ancestor and other diverse, less frequent biofilm-specialist mutants, resulting in populations that have differentiated to fill multiple niches within the biofilm life cycle. This work also demonstrates the power of distributed research networks for addressing complex biological questions while promoting scientific literacy to the public.

Despite being the dominant form of microbial life, our understanding of the ecology, evolution, and genetic controls of adaptation within biofilms is still developing. What we and many others have learned is that bacteria evolve rapidly and conspicuously, often by producing new colony morphologies, when selected to improve surface growth in experimental models as well as in clinical and environmental settings (1–5). This dynamic can be captured in simple experiments conducted by secondary school students that not only improve their learning experiences but also provide them the opportunity to contribute to cutting-edge scientific research (6–8).

We might expect that interactions in biofilms would be complex as cells engineer their environment by secreting polymers that bind them to a surface and protect them from stressors, competitors, and predators. Two overlapping processes can explain the origin and maintenance of phenotypic diversity within biofilms. The first is the environmental structure itself, which allows multiple phenotypically equivalent mutants to arise and coexist in different regions (9). The second is ecological interactions made possible by structure, for example, when an early adhering population facilitates the attachment of different types (10, 11). However, the genetic and phenotypic outcomes of these processes are less clear but broadly important in natural and engineered systems, such as wastewater treatment (12) and in the clinic (13). For example, high-biofilm-forming bacterial and fungal mutants often arise during chronic infections, are associated with increased resistance to antibiotics and immune phagocytes, and worsen patient outcomes (14–16). There is a need to broadly characterize the spectrum of genotypes and phenotypes that generate biofilm diversity. Experimental evolution of microbial populations under conditions favoring biofilm, combined with contemporary genomic analysis, can be a powerful screen of these traits.

Our laboratory previously developed an *in vitro* model to study evolution throughout the entire biofilm life cycle of surface attachment, biofilm assembly, dispersal, and re-colonization (2). In short, bacteria are cultured for 24 h in test tubes that contain growth media and a polystyrene bead, which serves as a surface for bacteria to colonize and form a biofilm. The biofilm-covered bead is then transferred to a new test tube with fresh media and a sterile bead. Beads are serially transferred to select for biofilm-adapted mutants that can disperse from the old bead and recolonize and assemble a new biofilm on the new bead every 24 h, yielding approximately 6.6 generations per day based on a population-wide 100-fold increase (2). This model has been used by us to better understand the dynamics of biofilm evolution and associated ecological diversification in multiple species (*Burkholderia cenocepacia*, *Acinetobacter baumannii*, and *Pseudomonas aeruginosa*) under various environmental stressors (e.g., nutrients and antibiotics) (2–4, 11, 17–23). The bead model has also been adapted by many others to address similar questions and is routinely paired with whole genome sequencing of mutant clones and evolved populations to identify causes of new phenotypes (24–26). A surprising finding from these studies is the extent of genetic parallelism rather than a diverse array of pathways to adaptation. Despite the intuition that the biofilm life cycle is both structured and heterogeneous, and the observation of various colony phenotypes, mutations in relatively few genes encoding major biofilm regulators accounted for most of the adaptations (21, 23).

One possible explanation for convergent evolution in the biofilm bead model is that each study was conducted by one or a few investigators using well-controlled conditions, thereby focusing selection on limited traits. Higher experimental replication by many different researchers could address this concern. For this reason and others related to improving learning and access to authentic research, we adapted the bead model for use in secondary school classrooms (6). In this research-education partnership, students work with the harmless plant probiotic bacterium, *P. fluorescens* strain SBW25, which is both a model of this environmentally relevant species and shares many orthologs with the opportunistic pathogen *P. aeruginosa*. This program is called EvolvingSTEM and now engages thousands of students a year in authentic microbiology research in grades 6–12 in more than 20 schools in several U.S. states. Previous research on *P. fluorescens* SBW25 identified biofilm-related adaptations to static culture conditions, where populations rapidly evolve to form a biofilm mat at the oxygen-rich, air-liquid interface (27). The evolved populations diversified into three distinct colony morphologies: (i) smooth and round (the ancestral phenotype), (ii) wrinkly, and (iii) fuzzy (28). It is this rapid evolution to produce pellicles that encouraged us to use *P. fluorescens* with our bead model to study its adaptation to biofilm growth with students.

The most conspicuous new colony morphology seen in previous experiments is the wrinkly phenotype. This phenotype commonly results from mutations in one of three genetic pathways*—wsp*, *yfiBNR* (or *aws*), and *morA* (or *mws*)—that regulate the production of a secondary messenger, bis-(3’−5’)-cyclic dimeric guanosine monophosphate (cyclic di-GMP) (29). Cyclic di-GMP controls biofilm formation by regulating the activity of several downstream genes and proteins (30). The production of cyclic di-GMP is regulated by the opposing activity of two enzyme families: diguanylate cyclases (DGCs) that synthesize cyclic di-GMP and phosphodiesterases (PDEs) that degrade it (30). High cellular levels of cyclic di-GMP are associated with surface attachment and biofilm production, while low levels are associated with increased motility, solitary behavior, and bacterial dispersal (31). Mutations in the *wsp*, *yfiBNR*, and *morA* pathways lead to constitutive activation of a DGC, which results in overproduction of cyclic di-GMP (29, 32), and, ultimately, increased biofilm formation through increased production of acetylated cellulose (33, 34). In contrast, fuzzy phenotypes are a result of mutations in *fuzY*, which encodes a β-glycosyltransferase predicted to modify LPS O-antigens. Mutations in *fuzY* are thought to promote cell-cell contact, but the mechanistic benefits of mutations in this gene are less well understood (35).

*P. fluorescens* populations evolved by high school students with our EvolvingSTEM evolution-in-action experiment often exhibit diverse colony morphologies within and among populations that resemble those seen under selection in static culture conditions (6). We wondered if the phenotypic similarities of mutants adapted to static culture and those in our bead model are caused by the same underlying genetic changes; hence, we sequenced 69 clones from populations almost entirely evolved by high school students. We found that *P. fluorescens* adapts to form biofilms on surfaces through mutations in some of the same genetic pathways as those used to form pellicles in unshaken vials and also uncovered novel biofilm adaptations. We then sought to identify what drives evolutionary dynamics in our bead model and whether these dynamics differ when bacteria are grown in subinhibitory levels of the common antimicrobial triclosan, which was used to prevent fungal and bacterial contamination at some schools. This led to the discovery that biofilm adaptation in our model is primarily driven by lineages that do not change colony appearance but acquire loss-of-function mutations in PFLU0185, a highly conserved gene across pseudomonads encoding a DGC and dominant PDE (36–39). Although PFLU0185 mutants share the same colony morphology as their ancestor, further phenotyping revealed adaptations in biofilm formation and motility. We therefore suggest renaming this locus *bmo*, for biofilm and motility regulator. Population sequencing, microscopy, and multi-genotype competition experiments suggest that *bmo* mutants coexisted with their ancestor and with rarer, genetically diverse lineages producing wrinkly and fuzzy colonies in dynamics that can be explained by multi-niche selection (40).

## RESULTS

### Student-led experiments rapidly uncover diverse phenotypes

**FIG 1:**
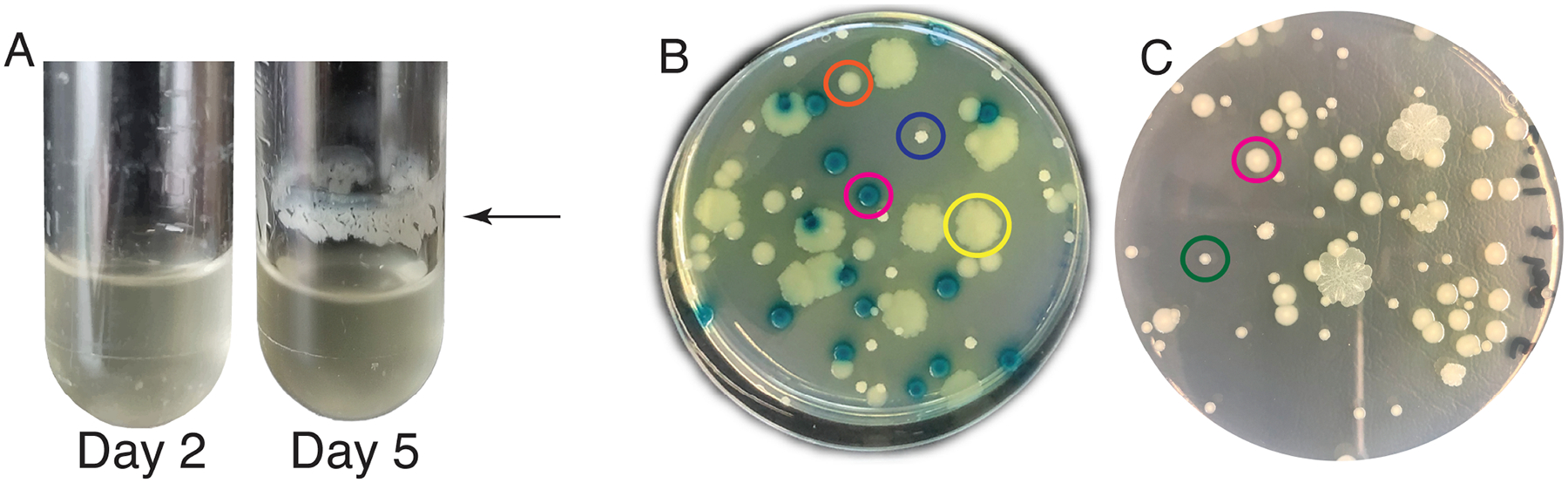
Biofilm growth patterns and colony morphologies of *P. fluorescens* SBW25 and evolved biofilm-adapted mutants. (A) Evolved populations often have a distinct ring of biofilm at the air-liquid interface. (B) A constructed population demonstrates commonly identified colony phenotypes: the *P. fluorescens* ancestor (lac+) has blue, smooth-edged circular colonies (pink circle), the *bmo* (PFLU0185) mutant has yellow, smooth-edged circular colonies (orange circle), the *fuzY* mutant has large, yellow, irregular colonies (yellow circle), and the *wspF* mutant has small, white, irregular colonies (purple circle). This constructed community is the one used in the experiments described below. (C) Student-led experiments also include populations with small colony variants (green circle) that can be distinguished from their ancestor (pink circle) by their significantly smaller size.

Our EvolvingSTEM evolution-in-action experiment allows students to observe bacterial populations evolving and diversifying within days as they adapt to a daily selection regime to complete the entire biofilm life cycle of dispersal, surface attachment, and biofilm maturation. Students work with a harmless plant probiotic bacterium, *P. fluorescens* strain SBW25. As the populations evolve, students can observe at least two types of phenotypic change. First, as high biofilm-forming mutants increase in frequency in the population, students often see a more robust layer of biofilm form in their test tubes at the air-liquid interface. Second, students can observe changes in the colony morphologies of bacteria grown on agar plates. While the ancestral population produces only smooth, circular colonies, evolved populations often exhibit diverse, conspicuous colony morphologies within and among populations (Fig. 1). The most commonly identified adapted colony morphologies in our bead-biofilm selection model include wrinkly and fuzzy morphologies that appear identical to those identified in previous studies that selected for *P. fluorescens* SBW25 biofilm mats under static culture conditions (28) (Fig. 1). In addition, our bead model often selects for small colony variants that can be distinguished from their ancestor by their much smaller colony size (Fig. 1) and increased uptake of Congo Red dye (Fig. S4), an indicator of increased polysaccharide production.

Students from three high schools—Winnacunnet High School (WHS, Hampton, NH, *N* = ~250 students), Pittsburgh Science and Technology Academy (ST, Pittsburgh, PA, *N* = 25 students), and Allderdice High School (A, Pittsburgh, PA, *N* = 24 students)—conducted experiments under slightly different conditions. Changes to the experimental protocol were made to accommodate class requirements and increase the accessibility of EvolvingSTEM in varied classroom settings. Our updated curriculum materials can be found at http://evolvingstem.org/curriculum/, which, in addition to the experimental protocol, includes a broad overview of the experiment, our NGSS-aligned (41) three-dimensional approach to curriculum development, materials and media needed to run the experiment, and helpful tips and tricks for educators. While WHS used standard aseptic technique, in other schools, we added the commonly used antimicrobial compound triclosan to the Queen’s B (QB) media and agar plates to broadly inhibit bacterial and fungal contamination. *Pseudomonas* species are known to tolerate or resist triclosan (42, 43), and this eliminates the need to use a flame in schools that do not have access to Bunsen burners and/or do not feel comfortable having students work near an open flame. Another difference was that WHS aerated growing bacterial cultures on a roller drum, which was not available in most classrooms; hence, we switched to using orbital shakers to aerate growing cultures.

Students plated samples of their bead-attached bacterial populations on agar at the beginning and end of the experiment. Ancestral *P. fluorescens* clones produce only smooth, round colonies, and this was the only morphology identified on the early plating day. Most bead-adapted populations contained wrinkly colony variants on the final plating day, and some had fuzzy and small colony variants (Fig. 1). Wrinkly and fuzzy colony variants had a similar appearance to those identified when *P. fluorescens* is under selection for the formation of biofilm mats in static culture conditions (27). While our experimental design also selects for biofilm-adapted mutants, it differs from static selection experiments in that bacteria must colonize a new surface each day, completing the entire biofilm life cycle of dispersal, attachment, biofilm assembly, and matrix building. We therefore explored if the convergent colony phenotypes we identified in these experiments were the result of convergent mutational targets by sequencing the genomes of wrinkly and fuzzy clones from independent populations. In addition, we sequenced the genomes of smooth colonies, which were identical in appearance to the ancestor, and small colony variants, which were the only other unique morphology noted in these experiments. In all, we sequenced 69 clones from 54 independent populations (Fig. 1; Table S1). From the 54 populations reported in this study, all but three contained wrinkly colonies, fuzzy colonies were identified in two populations, and small colonies were identified in five populations, although these frequencies are skewed toward colonies chosen from populations with visibly new morphologies. This collection of mutants was obtained from three different high schools and two different labs: three clones were sequenced from one population at Allderdice; 22 clones were sequenced from 10 populations at SciTech; 40 clones were sequenced from 39 populations at Winnacunnet over the course of 3 years; four clones were sequenced from four populations at university labs (three from our lab and one from Dr. Paul Turner’s lab at Yale).

### Biofilm adaptations include mutations affecting cyclic di-GMP production, LPS O-antigen structure, and periplasmic protein folding

Two remarkable properties of this collection of mutant genomes deserve highlighting before we explore the genes affected or their functional implications. First, in 53 of 69 mutants, only one mutation was identified, meaning that this single mutation experienced strong selection to rise to a detectable frequency and, in most cases, change the colony morphology (Table 1; Table S1). Consequently, the biofilm bead model serves as a potent forward genetic screen for single mutations that influence biofilm-related fitness. Of the remaining 16 mutants, 14 had two mutations, and two had three mutations. Second, the extent of site-specific parallelism in these mutants is extraordinary: 14 individual mutations occurred more than once in different clones. Although it is possible that a few instances of parallel mutations were not entirely independent (e.g., ones identified from the same school and year), most clearly arose independently from different schools and years. Exceptional examples include one SNP in *wspF* that was observed in six clones and another 33 bp deletion in *yfiR* observed five separate times (Table 1). This observation supports previous findings that revealed that mutational routes to wrinkly spreaders in *P. fluorescens* SBW25 are predictable based on genetic architecture, an extremely strong selective advantage for certain mutations, and locally higher mutation rates in some genes (44). Overall, 23 unique genes became mutated and were affected by a roughly equal mix of small insertion/deletions (indels) and SNPs, with fewer larger deletions. This spectrum of mutations demonstrates that *P. fluorescens* experiences strong selection for a narrow set of traits in the biofilm bead model that often favors identical mutations in independent experiments.

**FIG 2:**
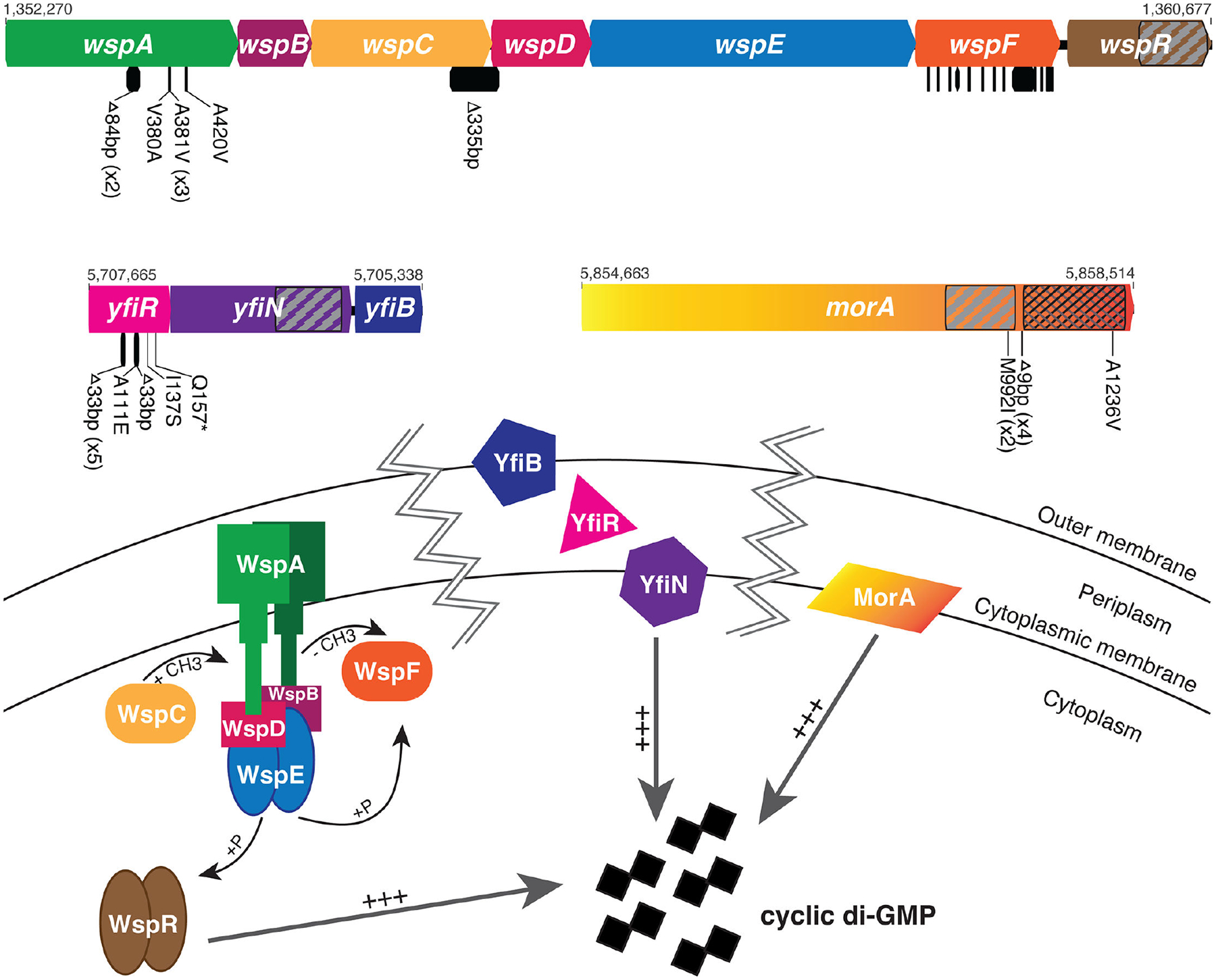
Wrinkly colony phenotypes are caused by mutations in the *wsp*, *yfiBNR*, and *morA* pathways. Mutations are shown as black bars below the gene diagrams. When multiple independent mutations occurred, their counts are in parentheses. Mutations in *wspF* (31 in total) are too numerous to display. Diguanylate cyclase (DGC) domains are indicated with diagonal hatching; phosphodiesterase (PDE) domains are indicated with cross-hatching. Genome positions of the beginning and end of each operon/gene are indicated above the gene diagrams. Table 1 and Table S1 contain a complete list of mutations. All mutations are predicted to cause constitutive activation of a DGC, increasing cyclic di-GMP synthesis.

The genomes of 57 clones with wrinkly phenotypes were sequenced from 50 independently evolved biofilm populations. In agreement with previous studies on biofilm formation in static culture conditions, causative mutations were identified in the *wsp*, *yfiBNR*, and *morA* pathways (Fig. 2; Table 1), which supports a critical role for these pathways in biofilm regulation under diverse selective regimes (i.e., as biofilm mats in static cultures and surface-attached biofilms). Mutations in the *wsp* pathway were the most common cause of wrinkly phenotypes across all sequenced clones, with approximately 70% having a single causative mutation in this pathway. Mutations were primarily found in *wspF* (PFLU1224, 33 clones), followed by *wspA* (PFLU1219, six clones). In addition, a single clone had a 335 base-pair deletion that overlapped the coding regions of *wspC* (PFLU1221) and *wspD* (PFLU1222), resulting in a WspC-WspD fusion protein. The *yfiBNR* pathway was the second most common cause of wrinkly phenotypes, with approximately 16% of clones having a single causative mutation in *yfiR* (PFLU5211, nine clones). Mutations in *morA* (PFLU5329, five clones) were the least common cause of wrinkly phenotypes, with approximately 9% of clones having a single causative mutation. Surprisingly, all three clones isolated at Allderdice had mutations in two pathways. These clones contained a single, nonsynonymous SNP in the PDE domain of *morA*. One clone had an additional nonsense mutation in *wspF*; one had an additional missense mutation in *wspA*; and one had an additional deletion in the linker region between the DGC and PDE domains of *morA*.

**FIG 3:**
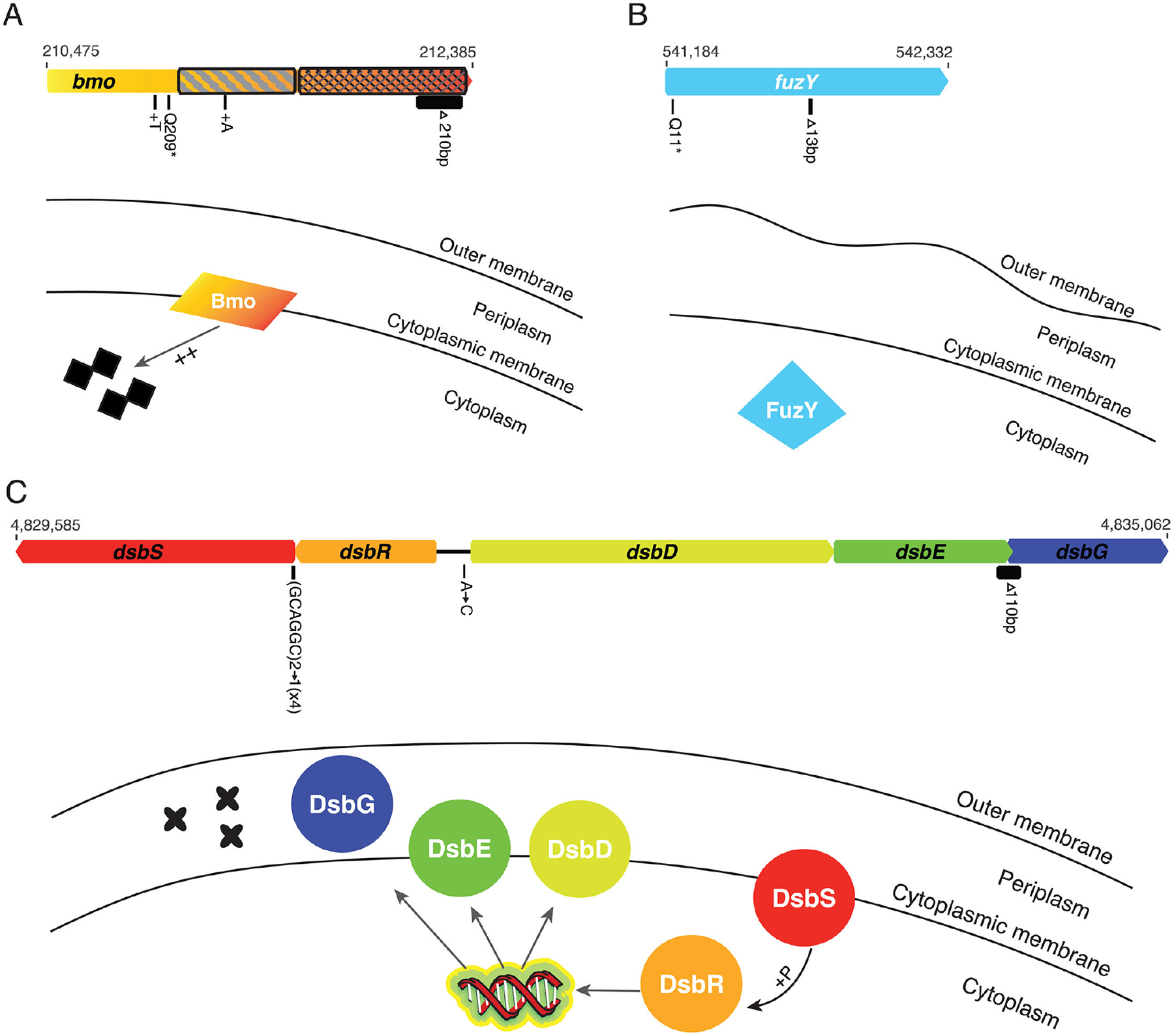
Biofilm adaptations caused by mutations in LPS, disulfide bond formation, and a novel regulator of cyclic-di-GMP. Mutations are shown as black bars below the gene diagrams. When multiple independent mutations occurred, their counts are in parentheses. DGC domains are indicated with diagonal hatching; PDE domains are indicated with cross-hatching. Genome positions of the beginning and end of each operon/gene are indicated above the gene diagrams. Mutations in *bmo* (PFLU0185) are predicted to increase basal levels of cyclic di-GMP (A). Mutations in *fuzY* are predicted to modify LPS O-antigens (B). Mutations in *dsb* genes are predicted to cause misfolded periplasmic proteins (C). Table 1 and Table S1 provide mutation details.

Fuzzy mutants were identified in evolved populations at several schools, although less frequently than wrinkly mutants. While we did not sequence any fuzzy mutants from high school experiments, two fuzzy mutants isolated in the laboratory had predicted loss-of-function mutations in *fuzY* (PFLU0478) (Fig. 3). In addition, we sequenced the genomes of six small colony variants isolated by students and in the laboratory. These mutants contained single mutations in a predicted two-component system sensor histidine kinase, *dsbS* (PFLU4380), or in the operon it is predicted to regulate, *dsbD* (PFLU4382)-*dsbG* (PFLU4384) (Fig. 3). Four clones had an in-frame deletion in the N-terminal region of *dsbS*, one clone had a 110 base-pair deletion that overlapped the coding regions of *dsbE* (PFLU4383) and *dsbG*, resulting in a DsbE-DsbG fusion protein, and one clone had a mutation in the intergenic region upstream of *dsbR* (PFLU4381) and *dsbD* (Fig. 3). Finally, we also sequenced clones from five evolved populations that retained the ancestral smooth morphology. Although one smooth clone had no mutations, four clones had a mutation in a gene encoding both DGC and PDE domains, PFLU0185, and this was the only mutation identified in three clones (Fig. 3). We have named this gene *bmo* for its roles in biofilm formation and motility, which are further described below.

### Strong selection for mutations in *bmo* during adaptation to the experimental biofilm life cycle

**FIG 4:**
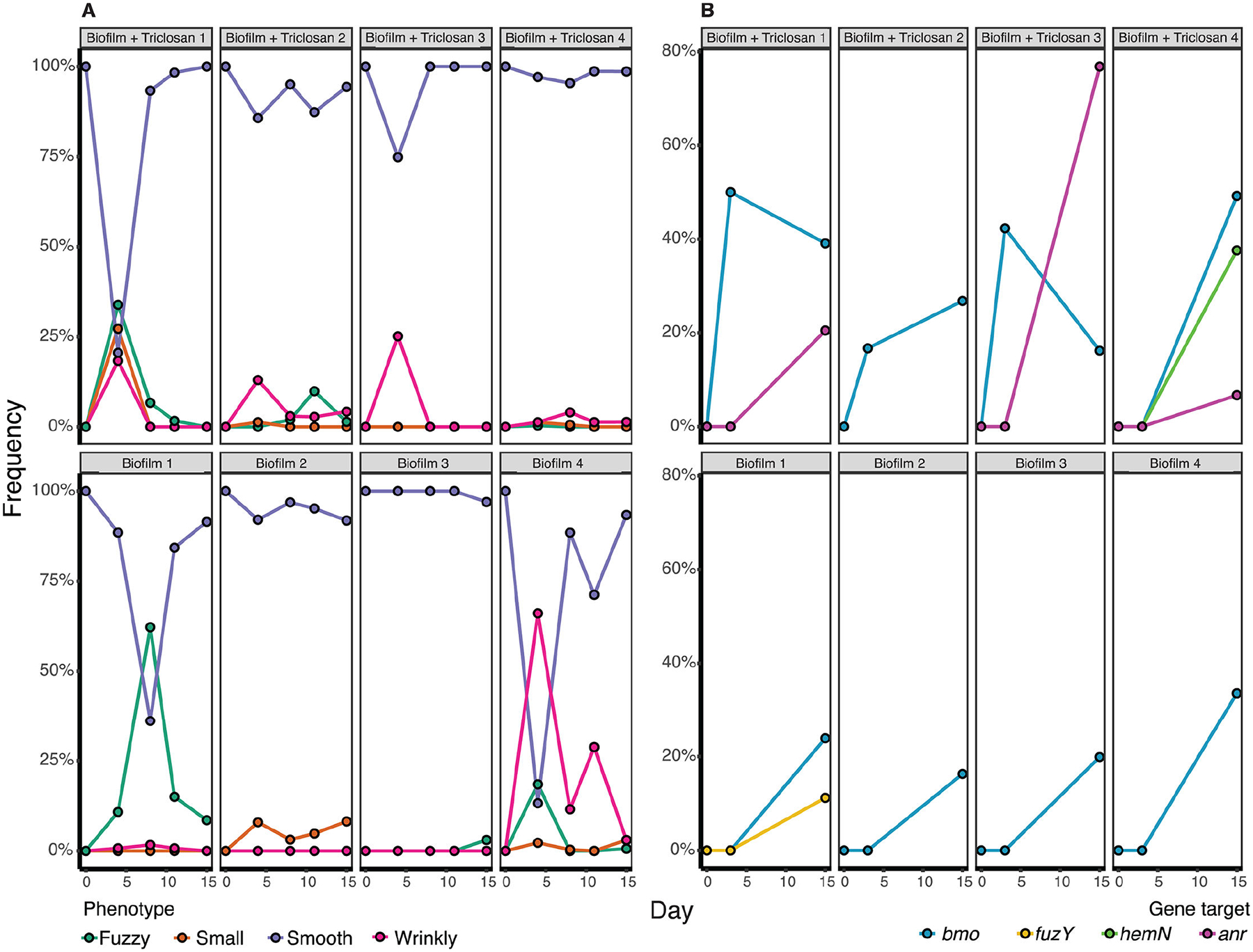
Dynamics of evolved phenotypes and genotypes during 15 days of experimental biofilm selection. Populations were grown for 15 days in QB media with or without triclosan with four plastic beads and passaged every 24 h by transferring one bead into fresh media with three new beads. Bead-attached populations were plated on days 4, 8, 11, and 15 and contained colonies with fuzzy, small, smooth, and wrinkly phenotypes (A). Whole-genome, whole-population sequencing was performed on days 3 and 15. Points include the cumulative frequency of mutations identified in each gene (B). A complete list of mutations can be found in Table S2.

We were intrigued by the discovery of mutations in a DGC-PDE encoding gene that did not change the mutant colony phenotype. Although an objective of the Evolving-STEM program is to visually demonstrate adaptation by natural selection by observing novel colony phenotypes after selection for improved biofilm formation, in fact, in most populations, the majority of colonies retain the smooth appearance of the ancestor after one week of passage. This suggests that focusing only on colony variants may miss biofilm adaptations. To address this limitation, we conducted a 15-day evolution experiment in which we used whole-population, whole-genome sequencing to identify all mutations reaching appreciable frequency in biofilm populations, regardless of whether they alter colony appearance. We also replicated this comparison in media containing triclosan, which we use in classrooms to guard against contamination and which appears to accelerate rates of diversification. Specifically, four replicate biofilm populations were propagated with and without 25 μg/mL triclosan for 15 days. We plated the bead-attached portion of each population after 4, 8, 11, and 14 transfers to observe phenotypic changes to population colony morphologies over time and performed whole-population, whole-genome sequencing after 2 and 14 transfers (Table S2; Fig. 4). In total, this experiment allowed for approximately 93 generations. We noted three distinct colony morphologies, wrinkly, fuzzy, and small, in addition to the ancestral, smooth phenotype. Biofilm populations grown in triclosan had moderately increased phenotypic diversity on the earliest plating day, but this diversity decreased over the course of the experiment, with all populations having over 95% smooth colonies by the final plating (Fig. 4A). Populations that were grown without triclosan maintained slightly higher diversity over time, and all populations had between 90% and 95% smooth colonies by the final plating (Fig. 4A).

Surprisingly, mutations in the *bmo* locus were the only gene-level parallel genetic change in all biofilm populations, and they rose to detectable frequencies by the third day of the experiment in media containing triclosan, but not in media without triclosan (Fig. 4B; Table S2). Multiple independent mutations in *bmo* were present within each of the three biofilm populations with added triclosan at day 3 at 16.7%, 42.3%, and 50% cumulative frequency, and by day 15, all eight biofilm-adapted populations had *bmo* mutants at >16% cumulative frequency (Fig. 4B). The only other notable biofilm-related mutation that we observed was in *fuzY*, which reached a frequency of 11.2% in one population that was grown without triclosan (Fig. 4B). Furthermore, three of the four populations grown with added triclosan gained mutations in a transcription factor, *anr* (PFLU4570), and one of the genes it regulates, *hemN* (PFLU4568) (Fig. 4B). These mutations may be adaptations to growth in triclosan and will be the subject of future study.

### Mutants are differentiated in biofilm phenotypes, including attachment, assembly, and motility

We predicted that mutations in *bmo* achieved the highest frequencies and repeatedly evolved in our biofilm selection model because they are best adapted to the cyclical requirements to attach to the plastic bead, assemble biofilms, and then disperse to colonize a new bead. We therefore characterized the motility, biofilm production, and cyclic di-GMP production for a representative *bmo* loss-of-function mutant with a single nucleotide insertion that results in a premature stop codon, truncating both the DGC and PDE domains (Table 1). We compared its biofilm-associated phenotypes to a representative wrinkly (*wspF* Q185*) and fuzzy (*fuzY* Q11*) mutant, along with the *P. fluorescens* SBW25 ancestor from which these mutants evolved.

**FIG 5:**
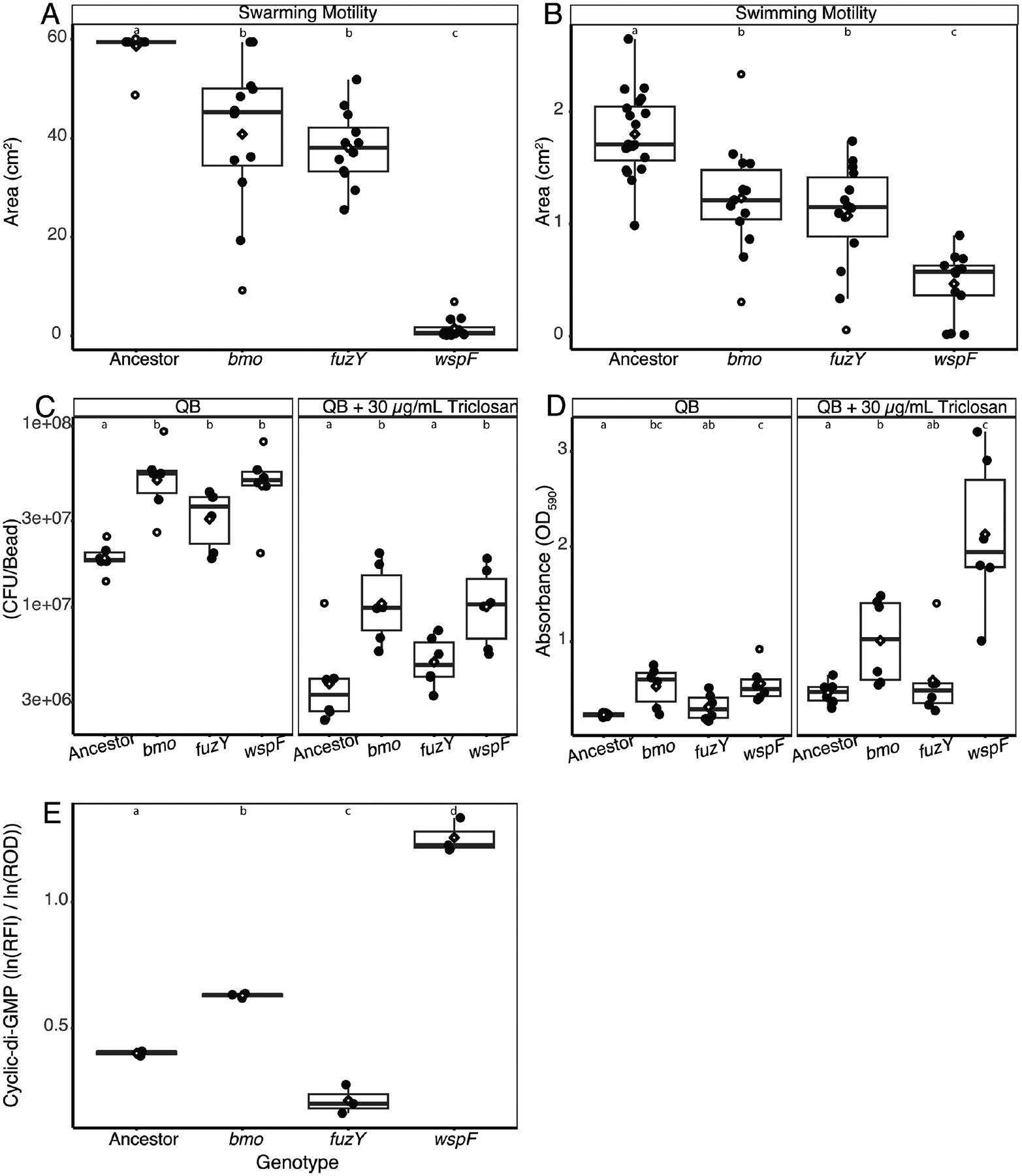
Biofilm-related phenotypes of representative *bmo* (D180*), *wspF* (Q185*), and *fuzY* (Q11*) *P. fluorescens* mutants in comparison to their ancestor. Swarming motility (A) and swimming motility (B) are reduced in comparison to the ancestor. Biofilm production measured by attached colony-forming units (C) and by crystal violet absorbance at 590 nm (D). Points are the average of three measurements recorded for each replicate. The results in QB media are similar but more pronounced with the addition of triclosan. Relative cyclic di-GMP production from a fluorescent reporter (E) is reported as ln(RFI)/ln(ROD600), where RFI is relative fluorescence intensity, and ROD600 is relative optical density. In all panels, mean values are indicated by diamonds, and outliers are indicated by an open circle. Statistical comparisons were conducted by one-way ANOVA with a Games-Howell post hoc test with *P* = 0.05, with letters at the top of each panel indicating statistically distinct groupings.

We first compared three common types of bacterial motility—swarming, swimming, and twitching. Swarming motility is movement across a semisolid surface that involves flagellar motility and biosurfactant production. Swimming motility is movement in liquid or low-viscosity conditions that requires functional flagella. Twitching motility is movement across solid or semisolid surfaces that requires the presence of retractile type IV pili. All biofilm-adapted mutants had reduced swarming and swimming motility in comparison to the ancestor (Fig. 5A and B). *wspF* mutants were the least motile, while *fuzY* and *bmo* mutants had intermediate phenotypes. Twitching motility was not displayed by any biofilm-adapted mutants or the ancestor, confirmed by comparison to *P. aeruginosa* PAO1, which possesses twitching motility (Fig. S1).

To estimate biofilm production by each mutant, we adapted the traditional crystal violet 96-well plate assay (45) to better align with how bacteria form biofilms in our bead model. Bacteria were cultured for 24 h in test tubes containing media and two plastic beads. We quantified total biofilm production by staining one bead with crystal violet, removing excess stain by washing the bead in water, and solubilizing biofilm-bound crystal violet by vortexing the bead in solvent. We also quantified biofilm population size by counting cells attached to the second bead by vortexing and dilution-plating. When grown in QB medium, *bmo* and *wspF* mutants have the highest total biofilm production and population size (Fig. 5C and D). One caveat to note is that *wspF* mutant populations often acquire compensatory, suppressor mutations that can rise to high frequencies in overnight cultures; hence, between 20% and 40% of the colonies counted had a smooth, ancestral phenotype. When grown in media with added triclosan, total biofilm production is doubled in *bmo*, *fuzY*, and the ancestor and quadrupled in *wspF* (Fig. 5C and D). Moreover, the population size of all genotypes is less than half that observed in triclosan-free media (Fig. 5C and D). Planktonic growth is also slower when triclosan is included in the media, and the final population sizes are smaller (Fig. S2).

To determine how these biofilm-adapted mutations affect cyclic di-GMP metabolism, we used a GFP reporter plasmid that responds to the intracellular concentration of cyclic di-GMP to estimate these changes. Levels of cyclic di-GMP differed significantly among *P. fluorescens* mutants following 24 h of growth in QB media (Fig. 5E). Cyclic di-GMP concentrations were highest for *bmo* and *wspF* mutants, with *wspF* producing nearly double the amount of cyclic di-GMP as *bmo*. Surprisingly, the *fuzY* mutant produced approximately half as much cyclic di-GMP as the ancestor, although this gene is not reported to have a role in the regulation of cyclic di-GMP. Taken together, these results demonstrate that *bmo* regulates biofilm formation and motility, and the loss-of-function mutations in this gene allow for the optimization of biofilm production and planktonic growth in our bead model.

**FIG 6:**
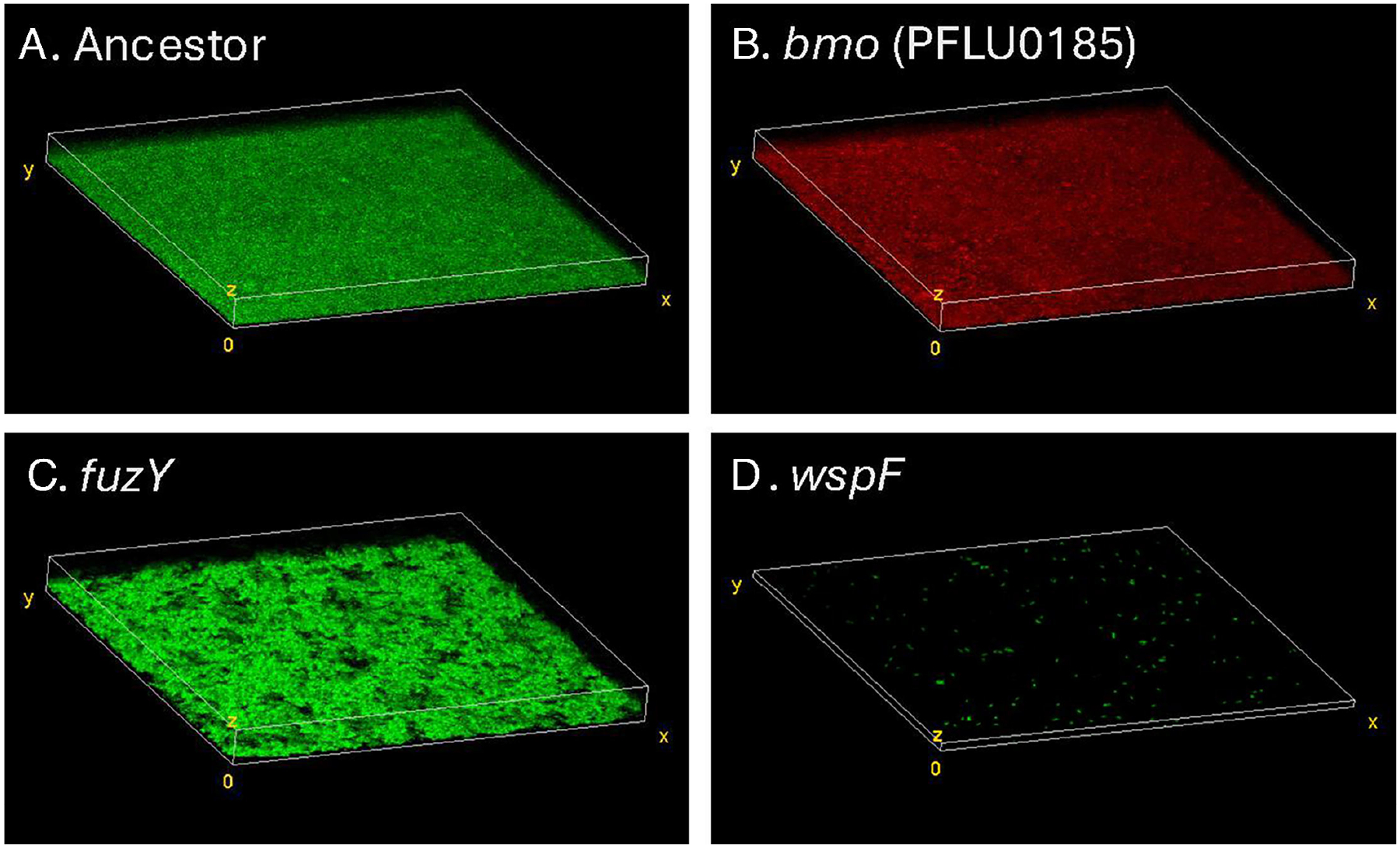
Confocal microscopy of representative *P. fluorescens* mutants shows differentiated biofilm phenotypes. Ancestor (A), *fuzY* (C), and *wspF* (D) are chromosomally tagged with eGFP, and *bmo* (B) is chromosomally tagged with mCherry. Both Ancestor and *bmo* exhibit uniform biofilm structure with similar properties. The *fuzY* biofilm is composed of many aggregates and is thicker. The *wspF* biofilm bacterial cells were aggregated in small clusters that were sparsely attached to the glass surface, but we identified large aggregates in liquid media that were more likely to attach to plastic surfaces (not shown). Mixtures of these mutants were also imaged and are reported in Fig. S3.

We sought to understand how the different macroscopic and regulatory phenotypes of *P. fluorescens* mutants generated distinct microscopic biofilm phenotypes. We therefore used confocal microscopy to image biofilms produced by our representative *bmo*, *wspF*, and *fuzY* mutants as well as the ancestor that contained a constitutively active fluorescent reporter inserted at a neutral site on the chromosome. Both the ancestor and *bmo* formed relatively confluent biofilms of even thickness, and their similar appearances suggest that they would compete for the same niche space on the bead (Fig. 6). In contrast, the *fuzY* biofilm was more clumped and uneven in thickness and coverage, whereas the *wspF* biofilm was surprisingly sparse, with small, dense clusters (Fig. 6). It is notable that we imaged biofilms on glass slides rather than on the polystyrene beads on which these mutants evolved and that the supernatant of the *wspF* cultures contained large aggregates that did not adhere well to glass. To explore how these mutants interact in a diverse biofilm, we imaged mutants with different fluorescent markers that were co-cultured in pairs (Fig. S3). Overall, the observed patterns of biofilm assembly agreed with predictions from mutant growth alone, with interspersed and confluent growth by *bmo* and the ancestor but fewer discrete patches of different sizes and forms produced by *wspF* and *fuzY*. Taken together, these images indicate that biofilm mutants in different pathways are ecologically distinct and produce clusters or coatings of varying biomass and form, both alone and in mixture.

### Genetic diversity during biofilm selection is maintained by competition and niche differentiation

**FIG 7:**
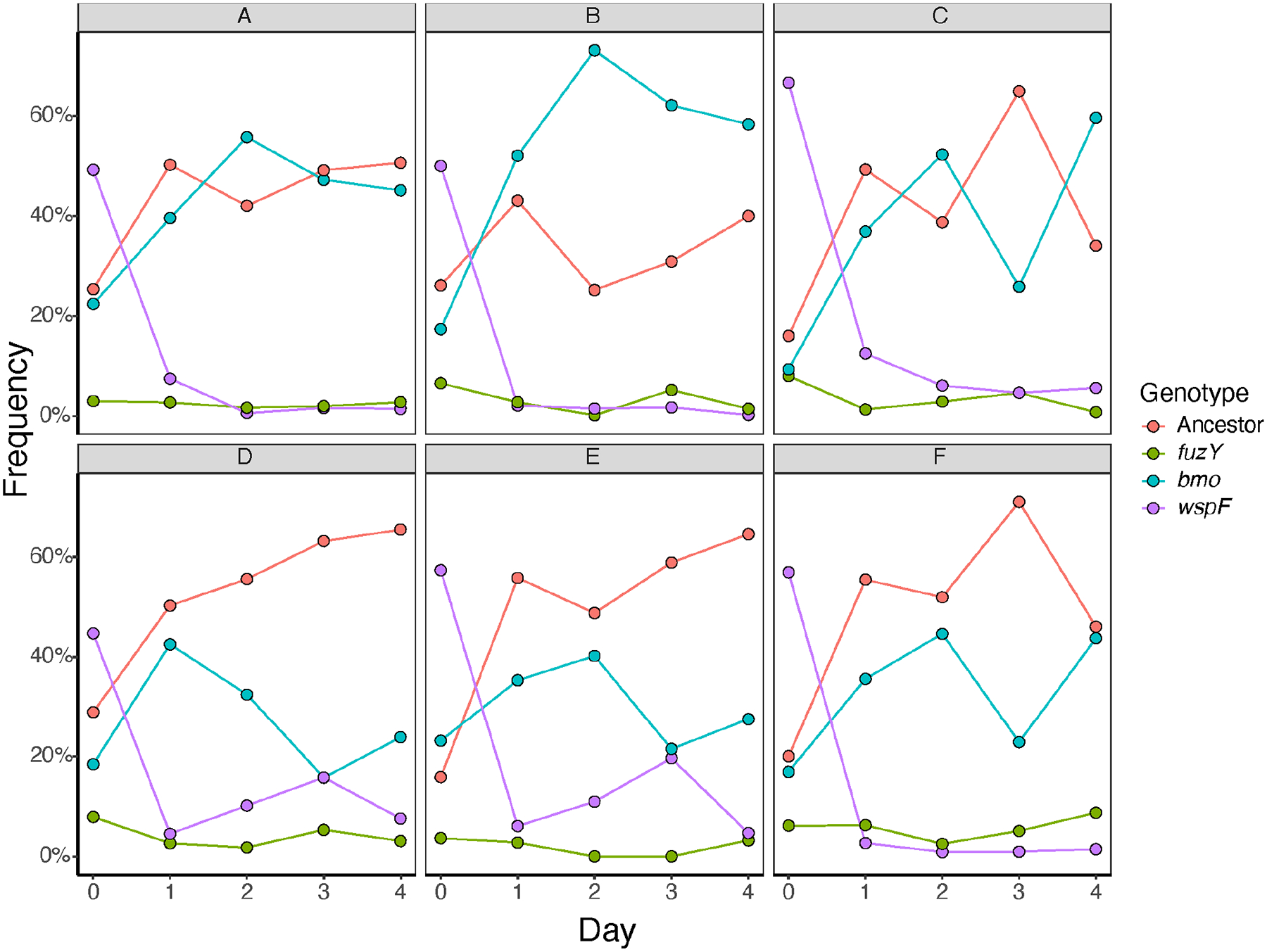
Ecological dynamics of constructed communities indicate multi-niche selection. Six replicate populations (panels A–F) containing representative, biofilm-adapted loss-of-function mutants in *bmo* (PFLU0185), *fuzY*, and *wspF* and the *P. fluorescens* ancestor from which they were evolved were propagated in the biofilm bead model and plated daily to assess frequencies. All mutants persisted. *WspF* declined rapidly in frequency, and *fuzY* was seeded at a relatively low starting frequency, and both of these mutants persisted at low frequencies throughout the experiment. After 24 h of growth, *bmo* and the ancestor both increased in frequency from their initially seeded frequency. Their populations then fluctuated at intermediate frequencies and sometimes opposing trajectories.

**FIG 8:**
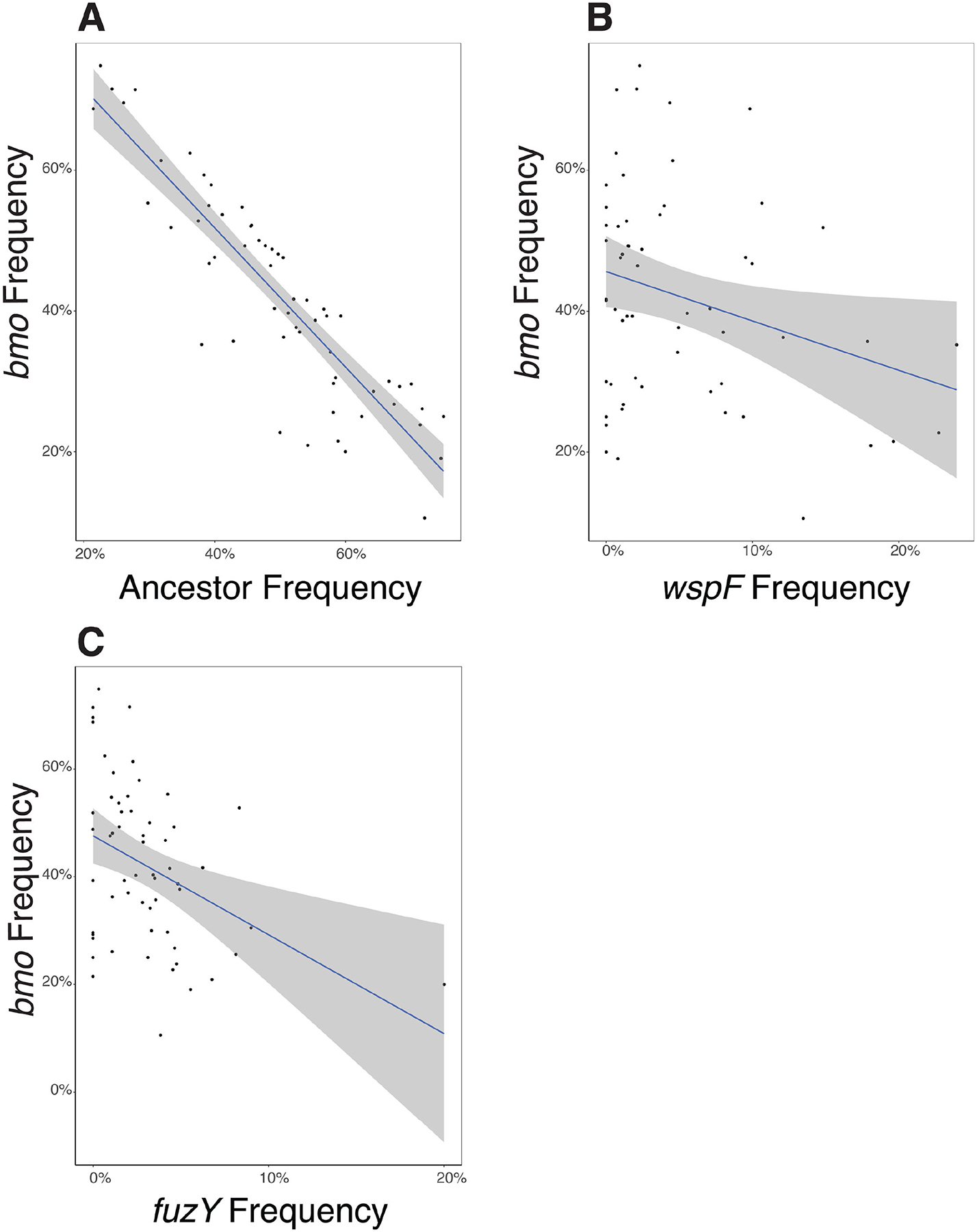
Linear regressions of *bmo* frequencies, as compared to the frequencies of the ancestor (A), *wspF* mutant (B), and *fuzY* mutant (C). Linear regressions of the *bmo* mutant in comparison with all other variants in our constructed communities (Fig. 7) found a significant, negative relationship between the frequency of the *bmo* mutant and all other genotypes, indicating that *bmo* simultaneously competes with all other variants in the population mixtures. Regression statistics are shown in Table S3.

An important and unusual finding from these studies is that *P. fluorescens* populations grown in the biofilm bead model rapidly and repeatedly diversify into multiple phenotypes and genotypes that coexist, with no genotype ever reaching 100%. This observation implies that selection acts strongly on mutants that inhabit different niches of varied capacity. This leads to the prediction that if putative niche specialists are mixed in equal amounts and propagated, their frequencies should equilibrate according to the capacity of each niche and the relative competitiveness of each mutant in conditions of niche overlap (e.g., growth on a common carbon source). We created a mixed population of the three representative biofilm-adapted mutants *bmo*, *wspF*, and *fuzY* and their *lac*-marked ancestor. This mixed population included a *bmo* loss-of-function mutant with a single nucleotide insertion that results in a premature stop codon truncating both the DGC and PDE domains, a *wspF* mutant (Q185*), and a *fuzY* (Q11*) mutant. We used this mixed population to inoculate six replicate cultures grown in our bead model of biofilm growth for 4 days without triclosan and plated daily to determine the frequency of each genotype on newly colonized beads (Fig. 7). As predicted, all genotypes coexisted within each population, with *wspF* and *fuzY* mutants generally at low frequencies, and the *bmo* mutant and ancestor at intermediate frequencies. In addition, frequencies of *bmo* and its ancestor were anticorrelated in half of the replicates, which suggests that they occupy differentiated but overlapping niches, as the microscopy indicated and ecological literature supports (Fig. 6) (46, 47). We used linear regression to test this hypothesis for all genotype combinations and found a significant, negative relationship for the *bmo* mutant frequency with frequencies of all other genotypes, which is consistent with competition (Fig. 8; Table S3). In summary, our model of the biofilm life cycle exerts strong, parallel selection for different phenotypes that balance biofilm production, growth, dispersal, and reattachment in different ways. Mutants adapted to different aspects of these traits can coexist by invading in conditions in which they are more fit, while declining in conditions where they are less competitive.

## DISCUSSION

### Student experiments discover new genetic pathways of adaptation to the biofilm life cycle

Involving secondary school students in distributed scientific experiments in their own science classrooms offers a high level of experimental replication that can broadly test a common hypothesis. Their experiments with the biofilm bead model selected for phenotypes that excel during daily cycles of dispersal, recolonization, and biofilm assembly. By picking many representative clones with diverse colony phenotypes and then genotyping them by whole-genome sequencing, we use experimental evolution as a powerful forward genetic screen. This approach identified mutants that support previous findings from pellicle selection experiments and also uncovered novel adaptive pathways within the biofilm lifestyle. These findings support the hypothesis that the biofilm life cycle of attachment, assembly, dispersal, and reattachment acts on traits governed by a few central regulators, leading to strong parallel evolution across experiments. They also reveal that different selective environments can uncover alternative adaptations in novel pathways to biofilm formation. Because most of these mutants contain only one SNP or small indel in their genome, the relationships between genotype and phenotype are direct. In this section, we discuss predicted molecular and biochemical effects of these mutants in detail, several of which strengthen or modify existing models of *Pseudomonas* biofilm gene regulation. We begin by analyzing mutations producing the well-known wrinkly and fuzzy colony morphologies and follow with a discussion of the potential causes of beneficial small colony variants.

### Student-selected colony variants support previous findings of genetic pathways important for biofilm formation

The most common cause of wrinkly phenotypes was mutations in the *wsp* pathway, a chemotaxis-like system composed of seven genes: *wspA-R*, under the control of a single promoter (Fig. 2) (48). WspA is a methyl-accepting chemotaxis protein that is anchored at the cytoplasmic membrane by the scaffolding proteins WspB and WspD. Upon sensing an environmental signal, conformational changes in the WspA-B-D complex result in WspA being methylated by WspC, a constitutively active methyltransferase. This begins a signaling cascade that activates WspE, a histidine kinase response regulator. WspE goes on to activate WspR, a DGC, through phosphorylation, increasing the levels of cyclic di-GMP and ultimately leading to increased biofilm formation through increased cellulose production (33, 34). WspE also phosphorylates WspF, a methylesterase that negatively regulates the system by demethylating WspA. In general, the Wsp pathway mutations we identified are predicted to cause constitutive production of cyclic di-GMP and, consequently, increased biofilm formation. We identified deletions in *wspA* that have been shown *in P. aeruginosa* to lock the pathway on in a methylation-independent manner (49), whereas nonsynonymous mutations in the *wspA* signaling domain likely disrupt the stability of its trimer-of-dimer interactions, increasing WspE activation (50, 51). The mutation producing a WspC-WspD fusion protein likely leads to hypermethylation of WspA because it physically links WspC to the WspA-B-D complex (52). The many different mutations in *wspF* include nonsynonymous substitutions, many of which occurred at the same site repeatedly, insertions, and deletions, including frameshift and nonsense mutations that introduced premature stop codons (Fig. 2). These mutations almost certainly interfere with WspF activation and/or its ability to demethylate WspA (49, 53).

The second most common cause of wrinkly phenotypes was mutations in the *yfiBNR* pathway, which is composed of three genes under the control of a single promoter (Fig. 2). YfiN is a DGC that is localized to the cytoplasmic membrane and negatively regulated by the attachment of the periplasmic YfiR protein (54, 55). In response to membrane stress, YfiB, a lipoprotein located at the outer membrane (55), adopts an elongated conformation, which allows it to bind to and sequester YfiR (56, 57). YfiN is therefore released from its negative regulation and able to catalyze cyclic di-GMP production, promoting biofilm formation (Fig. 2). The C-terminal region of *P. aeruginosa* YfiR is predicted to contain the attachment site to YfiN (55, 58); therefore, the *yfiR* missense and nonsense mutations we discovered in this region may eliminate YfiR’s ability to bind to YfiN. *P. aeruginosa* YfiR forms a dimer in its active form via salt bridges formed by R98 and D80 and hydrogen bonds between T76 (58). Notably, five *yfiR* mutants experienced the same 33 bp deletion from T75 to T85 in *P. fluorescens* that removes these homologous regions involved in dimerization. In addition, both this deleted region and the downstream 33 bp deletion contain or are located close to conserved cysteine residues that in *P. aeruginosa* form disulfide bonds important for proper protein folding (58). Deletions in these regions are therefore likely to lead to protein misfolding that inhibits YfiR’s ability to bind YfiN, thereby derepressing the DGC, which promotes a biofilm lifestyle.

Mutations in *morA* were the third most likely cause of wrinkly phenotypes. MorA is a single protein localized to the cytoplasmic membrane that contains two PAS sensor domains as well as active DGC and PDE domains; hence, it can catalyze both production and degradation of cyclic di-GMP (Fig. 2) (29, 59). We identified two identical nonsynonymous substitutions in the DGC, three mutations affecting the linker region between DGC and PDE domains that likely cause conformational changes that increase DGC activity (23), and three identical mutations in the PDE that appear to disrupt its activity (Fig. 2). The elevated biofilm of all these mutants indicates that their effects increase cyclic di-GMP levels.

Fuzzy colony phenotypes are caused by loss-of-function mutations in *fuzY* (Fig. 3). Although it is important to note that both of our sequenced mutants contained secondary mutations—one a synonymous mutation in PFLU2168, a hypothetical protein, and the other a nonsynonymous mutation in PFLU1660, a putative glycosyltransferase. We believe that the *fuzY* mutations are the most likely cause of the changes in colony phenotype because they share an identical phenotype to previously reported *fuzY* loss-of-function mutants (35) and other mutants in our collection did not display a fuzzy phenotype but did have the same mutation in PFLU1660. In *P. fluorescens* SBW25, *fuzY* is part of a five-gene operon that was predicted to encode a β-glycosyltransferase that plays a role in modifying lipopolysaccharides (LPS) O-antigens, the outermost portion of the LPS (35). The reciprocal best match of *fuzY* homologs in *P. aeruginosa* strain PAO1 is *wapH*, which participates in the synthesis of the LPS core (60). In either case, in addition to roles in pathogenesis and symbiosis, O-antigen structure plays an important role in bacterial adhesion and cohesion (61). In *P. aeruginosa*, O-antigen mutants exhibit increased cell-cell cohesion and increased adhesion to glass surfaces (62). In our bead selection experiment, we expect that mutations in *fuzY* are beneficial because they increase both cell-cell and cell-surface contact, which would provide an advantage in adhering to the surface of the bead.

### Novel biofilm adaptations associated with mutations predicted to disrupt periplasmic protein folding

Biofilm evolution experiments with our bead model often select for small colony variants with increased Congo Red binding, which is consistent with higher cellulose production (Fig. 1; Fig. S4). In all instances, these phenotypes were the consequence of mutations in a pathway that regulates the formation of disulfide bonds, *dsbRS* and *dsbDEG*, which is critical for some proteins to be properly folded into their active state conformation (Fig. 3). Most knowledge of enzymes involved in the formation of disulfide bonds comes from studies in *Escherichia coli* (63, 64). In *E. coli*, the periplasmic oxidoreductase DsbA catalyzes the formation of disulfide bonds but is thought to perform this reaction indiscriminately by introducing bonds between neighboring cysteines (63, 65). Proteins that require disulfide bond formation between nonconsecutive cysteines undergo further processing through the activity of disulfide isomerases (DsbC and DsbG) that rearrange these bonds (66–68).

In *P. aeruginosa*, homologs of the regulatory two-component system *dsbRS* have been shown to regulate the activation of *dsbDEG* homologs partly by binding copper ions (69). Based on this study, we predict that evolved *dsbS* mutants lead to altered phosphorylation of *dsbR*, which interferes with its regulation of *dsbDEG*. The evolved intergenic mutation is found in the predicted DsbR binding site and would therefore reduce *dsbDEG* transcription. The evolved DsbE-DsbG fusion mutant likely interferes with the ability of these proteins to fold properly, with the downstream consequence of misfolded periplasmic proteins. Taken together, we predict that all mutations in this pathway result in misfolded periplasmic proteins, although the exact proteins that are affected are unknown. Interestingly, in *P. aeruginosa*, deletion of *dsbA* led to a small colony variant phenotype that appeared identical to that of our *P. fluorescens* mutants and was hypothesized to be a result of YfiR misfolding due to its cysteine residues that contribute to dimerization, resulting in constitutive activation of YfiN and elevated cyclic-di-GMP (55). However, the small colonies produced by *dsb* mutations differ phenotypically from the wrinkly mutants caused by *yfiR* mutations, suggesting different effects on biofilm requiring further study.

### Discovery of frequent mutations in phosphodiesterase bmo that do not change colony morphology

Clones retaining the ancestral, smooth colony phenotype at the end of evolution experiments often acquired a mutation in PFLU0185/*bmo*, a gene with both DGC and PDE domains that is highly conserved in the genus *Pseudomonas* and predicted to function primarily as a PDE (Fig. 3) (38, 39). A representative mutant showed intermediate levels of both biofilm production and motility, leading us to name this gene *bmo* for its role in balancing both biofilm and motility. We predict that our model selects for *bmo* mutants because they act as biofilm generalists that have improved attachment and biofilm formation compared to their ancestor while also maintaining the ability to disperse from the old bead to recolonize the new bead. Characterization of multiple biofilm-associated phenotypes supports this hypothesis. Compared to the ancestor and the hyper-biofilm *wspF* mutant, *bmo* has intermediate motility, cyclic di-GMP, and overall biofilm attachment phenotypes, whereas its cellular attachment to the bead is as high as *wspF* (Fig. 5). Moreover, *bmo* biofilm phenotypes are nearly identical to the LPS mutant *fuzY*, although *bmo* has somewhat improved motility, attachment, and overall biofilm formation, along with significantly greater cyclic di-GMP levels. It is also notable that *bmo* mutants appear to be selected more rapidly and at a higher frequency in media containing triclosan (Fig. 4), suggesting that the stress imposed by this compound interacts with the *bmo* regulatory pathway that remains to be defined.

*P. aeruginosa bmo* homologs have been shown to influence biofilm formation and regulate basal levels of cyclic di-GMP. A deletion mutant of the *P. aeruginosa* PA14 homolog (PA14_03720) had reduced swimming motility and increased twitching motility in comparison to wild-type PA14 (70). Deletion mutants of the *P. aeruginosa* PAO1 homolog (PA0285, *pipA*) have increased biofilm formation, aggregation, Psl production, and cyclic di-GMP production, and decreased swimming motility (36, 37, 39). A recent study that compared deletion mutants of *bmo* homologs in *P. aeruginosa* strains PAO1, PAK, and PA14, and *P. putida* strain KT2440 found that these mutants had increased early attachment (at 4, 6, and 8 h) in comparison to their wild-type counterparts and that the magnitude of these differences depended on strain background (39). Among the 22 unique mutants detected by deep sequencing of populations evolved in the bead biofilm model, most are predicted to (i) cause loss of function by introducing premature stop codons or (ii) negatively affect PDE function or PAS domain signaling through nonsynonymous SNPs (Table 2). We predict these mutants resemble the representative mutant clone we studied (Fig. 5) by having a higher basal level of cyclic di-GMP that improves their attachment to the bead surface and biofilm formation.

### Phenotypic differentiation among mutants reflects niche differentiation that enables coexistence

The spectrum of biofilm phenotypes arising in these experiments suggests that they exploit different ecological conditions, which could explain both their rapid emergence and subsequent coexistence. This model was supported by confocal microscopy of biofilms produced by individual mutants and mixed pairs (Fig. 6; Fig. S3), but these measurements were on glass rather than polystyrene and did not allow mutants to reach equilibrium frequencies. These glass-attached biofilms were also too variable to estimate reliable quantitative differences in biofilm properties that would support functional differentiation. Therefore, as a complementary assay of niche differentiation, we mixed representative mutants of each morphology and tracked their frequencies over a week of passage. The *bmo* mutant and the ancestor appeared to compete as their intermediate frequencies oscillated, whereas *wspF* and *fuzY* mutants rapidly declined but were maintained at low (~5%–10%) frequencies (Fig. 7). The cycling between the *bmo* mutant and the ancestor can be explained by their occupying overlapping but distinct niches (40, 47), whereas the persistence of *wspF* and *fuzY* at lower frequencies suggests they inhabit more differentiated biofilm niches but are outcompeted by the other genotypes under most conditions (Fig. 6; Fig. S3). These dynamics agreed with the phenotypic dynamics from the 15-day evolution experiment (Fig. 4), in which biofilm-adapted mutants with distinct wrinkly and fuzzy colony morphologies rapidly arose and persisted at low frequencies while ancestral colony morphologies consistent with persistence of the ancestor and biofilm-adapted *bmo* mutants predominated.

These overall interactions conform to a model known as multi-niche selection (71), which requires that each species has a selective advantage in some habitats, while others are superior in other habitats, with areas of niche overlap. We suggest that the repeated emergence and maintenance of genotypes with differentiated niches within the biofilm life cycle provides a clear example of how variable environments can favor balanced polymorphisms (2, 72). Given the ubiquity of microbial biofilms undergoing regular cycles of attachment, aggregation, dispersal, and reattachment, we can expect genetic and phenotypic heterogeneity like that shown here to be widespread. This diversity may therefore be expected in biofilms produced by natural populations of *P. fluorescens* in and around plants or in those produced by its relative *P. aeruginosa* in the built environment or when establishing opportunistic infections.

The mutants that are selected in our bead model reinforce and extend our understanding of how *P. fluorescens* and its pathogenic relative *P. aeruginosa* adapt when biofilm formation is advantageous. Mutations in the *wsp* cluster, in *yfiBNR*, and in *morA* are commonly recovered in infections caused by *P. aeruginosa* (54, 73–75), and their recovery here indicates their centrality to this lifestyle switch. In addition, our discovery of mutants in *fuzY* adds to evidence that LPS alterations enhance aggregation and potentially alter susceptibility to bacteriophage (35), and mutants in the *dsb* pathway and *bmo* highlight less understood genetic pathways to biofilm production that warrant more research into how *Pseudomonas* senses surfaces and initiates attachment.

## MATERIALS AND METHODS

### School bacterial growth conditions

Students cultured *P. fluorescens* strain SBW25, a plant-colonizing bacterium that was isolated in 1989 from the leaf surface of a sugar beet plant grown at the University Farm (Whytham, Oxford, UK) (Bailey & Thompson 1992; Rainey & Bailey 1996 Molecular Microbio). Students cultured bacteria in test tubes that contained 5 mL of Queen’s B growth media (6), Proteose Peptone No. 3, 1.5 g Potassium Phosphate Dibasic, 25 mL Glycerol, and 6 mL 1 M Magnesium Sulfate per liter) and a polystyrene bead and plated their bead-attached bacterial populations on Half-Strength Tsoy-Agar plates (15 g Tsoy and 15 g Agar per liter) at the beginning and end of the experiment. In addition, 25 μg/mL of the broad-spectrum antimicrobial triclosan was added to the media and agar plates used in the experiments at SciTech and Allderdice to inhibit bacterial and fungal contamination. *Pseudomonas* species are naturally resistant to triclosan, and this concentration was chosen because it is used in *Pseudomonas* isolation agar (e.g., Cat#17208 Pseudomonas Isolation Agar from Millipore Sigma).

WHS students grew *P. fluorescens* cultures for 13 days (~86 generations) at 28°C on a roller drum under biofilm selection conditions, which included seven bead transfers. SciTech and Allderdice students grew cultures for 4 days (~26 generations) on an orbital shaker at 150 rpm under biofilm selection conditions, which included three bead transfers. SciTech students grew their cultures at 28°C, while Allderdice students grew their cultures at room temperature because they did not have an available incubator.

### Genome sequencing and identification of mutants

Clones were selected from the final plating day of the EvolvingSTEM classroom experiment and grown overnight in 5 mL QB media. DNA was extracted from 1 mL of the overnight culture with the DNeasy Blood and Tissue Kit (Qiagen). Illumina whole-genome sequencing was performed by our laboratory to a minimum depth of 40× coverage on an Illumina NextSeq500 using the methods described in Baym et al. (76). Trimmomatic (77) was used to remove sequence adaptors, along with low-quality sequence fragments. We identified mutations by comparing evolved clones to the published genome of *P. fluorescens* SBW25 (NCBI Reference Sequence: NC_012660) using breseq (78).

The ancestral *P. fluorescens* SBW25 populations all exhibited a smooth colony morphology phenotype. In comparison to the published genome sequence, the ancestor used by all student populations had three intergenic mutations present in their ancestral populations: (i) +G at position 45881; (ii) +C at position 985333; and (iii) C_5→3_ at position 3447984. This ancestor was used in all phenotypic comparisons. The ancestor used by WHS in 2015 also had an additional 129 bp deletion at position 360368 and a 132 bp deletion at position 6223020.

### Experimental evolution and longitudinal whole-population sequencing

Populations were initiated with a single *P. fluorescens* SBW25 ancestral clone and evolved for 15 days (14 transfers) in 2 mL Queen’s B growth medium at 28°C on an orbital shaker at 150 rpm. Four replicate lineages were propagated under two biofilm conditions: biofilm selection + 25 μg/mL triclosan and biofilm selection without triclosan. Biofilm lineages were propagated using four beads each, following the methods described in previous studies (21, 22). After 24 h of growth, one bead was transferred to a new test tube with three new beads.

To perform whole-population sequencing, we collected bead-attached bacteria on days 3 and 15 by transferring two beads from each population to a tube with 1.2 mL PBS, which was vortexed for 2 min to release bacteria from the beads; 500 μL was immediately centrifuged for 10 min at 14,000 rpm, the supernatant was removed, and the pellets were stored at −20°C for DNA extraction. Sequencing libraries were prepared to a minimum read depth of 150× coverage and analyzed as above with the additional “-p” polymorphism flag in breseq to characterize all reliable mutations at intermediate frequencies in these populations.

### Motility assays

Assays were performed as described in Filloux and Ramos (2014) (79). Images were analyzed with FIJI (80). For swarming motility, we grew 6-h cultures in LB-Lennox Broth (10 g tryptone, 5 g yeast extract, 5 g NaCl per L) at 28°C, 150 rpm, then inoculated Swarming Motility Agar Plates (0.6% agar, 1× M8 solution, 0.2% glucose, 0.5% casamino acids, 1 mM MgSO_4_) with 2.5 μL culture pipetted onto the center of the plate, at the surface of the agar. Plates were incubated upright at room temperature (~22°C) for 16–24 h and photographed. For swimming motility, we grew 6-h cultures of *P. fluorescens* in LB-Lennox Broth at 28°C, 150 rpm, transferred 100 μL to a 1.5 mL microcentrifuge tube, dipped a 200 μL pipette tip into the culture, and used this to stab into the center of Swimming Motility Agar Plates (same recipe as Swarming Motility Plates, but with 0.3% agar). Plates were incubated upright at room temperature (~22°C) for 16–24 h and photographed. For twitching motility, we stabbed a match-head-sized inoculum from a 2-day-old streak plate into the center of an LB-Lennox Agar Plate (10 g tryptone, 5 g yeast extract, 5 g NaCl, 10 g agar, 0.5 g tetrazolium red, per L) all the way down to the plastic. Plates were inverted and stored in stacks of three or less in a plastic box lined with wet paper towels at 28°C, with the box lid slightly ajar for 24 h and photographed.

### Biofilm assays

Our biofilm assay is based on the bead model developed by our lab for studying biofilm formation and evolution. Bacterial cultures were inoculated in triplicate in 2 mL of Queen’s B media and two plastic beads (Cospheric, Polystyrene Spheres, 7.00 mm +/− 0.025 mm) and grown overnight at 150 rpm on an orbital shaker at 28°C. Each culture was sonicated for 10 s at 30% amplitude and used to inoculate two new cultures at a 1:100 dilution in QB, both with and without 30 mg/mL triclosan and two polystyrene beads. Cultures were then grown for 24 h on an orbital shaker at 28°C, 150 rpm, serially diluted, and plated. Biofilm formation was quantified by transferring one of the two beads to a single well of a 24-well plate with 1.5 mL of a 0.1% crystal violet solution and incubated at room temperature for 15 min. The bead was then washed three times in water for 1 min each time to remove excess crystal violet. Biofilm-bound crystal violet was then solubilized by vortexing the bead for 2 min in a 2 mL tube filled with 1 mL solubilization solution: 95% ethanol, 4.95% water, and 0.05% Triton X-100. The concentration of crystal violet was measured at an absorbance wavelength of 590 nm (OD590). Total colony-forming units per bead were determined by serial diluting and plating bacteria attached to the second bead. We adopted this method to (i) more accurately represent the conditions in which we grow biofilms in our experiments and (ii) allow us to differentiate between total biofilm production and bacterial attachment to the bead. As an indicator of EPS production, we plated mutants on half-strength T-Soy Agar plates (15 g Tsoy, 15 g agar, 0.02 g Congo red, 0.0075 g brilliant blue per liter) and visually assessed dye uptake, where dark red colonies indicate EPS overproduction.

### Intracellular cyclic di-GMP assay

The *P. fluorescens* ancestor and biofilm-adapted mutants were transformed with the cyclic di-GMP reporter plasmid, pCdrA-gfpC (Addgene plasmid #111614) (81). The reporter transcriptionally fuses the *P. aeruginosa* PAO1 *cdrA* promoter, which is responsive to cyclic di-GMP, to genes encoding green fluorescent protein. Starter cultures of each transformed *P. fluorescens* genotype and an untransformed ancestor control were grown in their own cultures of 2 mL of Queen’s B media at 28°C on an orbital shaker at 150 rpm for 24 h. Following this, experimental cultures containing 4 mL of Queen’s B media were inoculated with 10 μL of a given starter culture and incubated at 28°C on an orbital shaker at 180 rpm for 24 h. After incubation, a 100 μL sample of each experimental culture was used to capture fluorescence intensity and OD_600_. Cyclic di-GMP levels at 24 h were calculated by ln(RFI)/ln(ROD_600_), where RFI is relative fluorescence intensity (the ratio of absolute fluorescence intensity of experimental to control culture), and ROD_600_ is relative optical density (the ratio of absolute optical density at a wavelength of 600 nm of a bacterial culture to fresh media).

### Construction of fluorescently tagged P. fluorescens strains

A single colony of *P. fluorescens* was inoculated into 5 mL QB and grown overnight to stationary phase. One milliliter of culture was pelleted by centrifugation and washed twice with 300 mM sucrose. After resuspending the pellet in 100 μL of 300 mM sucrose, 5 μL pTNS3 and 5 μL of the desired pBT plasmid (Table S4) were added, and the suspension was incubated at room temperature for 5 min. The suspension was then transferred to an electroporation cuvette (2 mm gap) and pulsed at 2.5 kV. Cells were recovered in 1 mL LB, and transformants were selected on LB agar + 30 μg/mL gentamicin at 28°C.

### Confocal microscopy

*P. fluorescens* strains expressing eGFP or mCherry were pre-cultured for 24 h in glass-bottom plates containing 2 mL of Queen’s B media and shaken on an orbital shaker at a speed of 125 rpm. Biofilms generated by *P. fluorescens* were expected to attach to the surface of the glass. After pre-culture, the old media in the plates were removed by pipetting, and the biofilms were washed twice with 1 mL of PBS to remove excess planktonic bacterial cells. Following the washing step, 1 mL of fresh PBS was added to each glass-bottom plate to maintain the survival and limit the growth of bacterial cells within the biofilm. Images were captured at 400× magnification in both eGFP and mCherry channels on an Olympus FV-1000 confocal microscope. The Z-stack images spanned from the bottom to the top of the biofilms. Image processing was conducted using ImageJ (80) and BiofilmQ (82). The images shown are representative of multiple replicates but were sufficiently variable that we do not report quantitative measures of roughness, volume, and thickness from BiofilmQ, despite being consistent with the images shown.

### Multi-genotype competition assay

We created mixed populations of bacteria by combining a representative mutant of *bmo* (PFLU0185) (with a single nucleotide insertion resulting in a premature stop codon truncating both the DGC and PDE domains), wrinkly (*wspF* Q185*), and fuzzy (*fuzY* Q11*) mutants, along with a lac-marked *P. fluorescens* SBW25 ancestor. The *lacZ* marker is chromosomal and is neutral for fitness over the duration of these assays, as shown previously (83). All four clones were used to inoculate cultures containing 2 mL QB and two beads and grown overnight at 28°C on an orbital shaker at 150 rpm. Both beads were then transferred to 1.5 mL QB in small glass tubes and vortexed for 2 min, and this mixture was used to make freezer stocks. Bacteria from a portion of one freezer stock were diluted in QB, and 50 μL of this dilution was transferred to six replicate populations containing 2 mL QB and four beads. Starting frequencies of each mutant and ancestor were determined by diluting a spread-plating from each starting culture. Populations were then evolved for 4 days (three transfers) in 2 mL Queen’s B growth medium at 28°C on an orbital shaker at 150 rpm. Every 24 h, one bead was transferred to a new test tube with three new beads, and all newly colonized beads were vortexed for 2 min, diluted, and spread-plated to count changes in colony phenotypes over time.

## Supplementary Material

Supp evolved population genotypes **Table S2 (AEM02499-25-s0003.xlsx).** Population sequencing results from evolution experiment.

Supp genotype information **Table S1 (AEM02499-25-s0002.xlsx).** Genotypes of sequenced clones.

Supp Tables and Figs **Supplemental material (AEM02499-25-s0001.pdf).** Tables S3 and S4; Fig. S1 to S4.

## Figures and Tables

**TABLE 1:** Genotypes of *P. fluorescens* mutants determined by WGS that were isolated based on colony phenotype by students in different schools^a^

School (count)	Phenotype	Position (nt)	Mutation	Annotation	Gene
**ST**	**smooth**	**210961**	**+T**	**coding (565/1989 nt**)	***PFLU0185 (bmo)***
ST	smooth	211021	C→T	Q209* (CAG→TAG)	*PFLU0185 (bmo)*
ST^b^	smooth	211277	+A	coding (881/1989 nt)	*PFLU0185 (bmo)*
ST	smooth	212132	Δ210 bp	coding (1736–1945/1989 nt)	*PFLU0185 (bmo)*
ST/WHS^b^	wrinkly	1353115	Δ84 bp	coding (846–929/1623 nt)	*PFLU1219 (wspA)*
WHS	wrinkly	1353408	T→C	V380A(GTG→GCG)	*PFLU1219 (wspA)*
ST/WHS (2)	wrinkly	1353411	C→T	A381V (GCC→GTC)	*PFLU1219 (wspA)*
ST	wrinkly	1355373	Δ335 bp	fusion of 2 predicted ORFs	*[PFLU1221(wspC)]-[PFLU1222(wspD)]*
WHS	wrinkly	1358709	G→C	A32P (GCC→CCC)	*PFLU1224 (wspF)*
WHS (3)^b^	wrinkly	1358766	Δ15bp	coding (151–165/1011 nt)	*PFLU1224 (wspF)*
WHS (3)	wrinkly	1358780	(CTGATGGACCTGATC)_1→2_	coding (165/1011 nt)	*PFLU1224 (wspF)*
WHS	wrinkly	1358846	Δ18bp	coding (231–248/1011 nt)	*PFLU1224 (wspF)*
WHS	wrinkly	1358893	Δ27 bp	coding (278–304/1011 nt)	*PFLU1224 (wspF)*
WHS (3)	wrinkly	1358989	+A	coding (374/1011 nt)	*PFLU1224 (wspF)*
WHS	wrinkly	1359085	G→T	G157V (GGC→GTC)	*PFLU1224 (wspF)*
**ST**/WHS (2)^b^	**wrinkly**	**1359168**	**C**→**T**	Q185* (CAG→**TAG**)	***PFLU1224 (wspF)***
WHS	wrinkly	1359234	+G	coding (619/1011 nt)	*PFLU1224 (wspF)*
WHS	wrinkly	1359292	Δ150bp	coding (677–826/1011 nt)	*PFLU1224 (wspF)*
WHS	wrinkly	1359330	G→C	A239P (GCC→CCC)	*PFLU1224 (wspF)*
WHS (6)	wrinkly	1359364	G→C	R250P (CGG→CCG)	*PFLU1224 (wspF)*
WHS^b^	wrinkly	1359370	C→T	S252L (TCG→TTG)	*PFLU1224 (wspF)*
ST	wrinkly	1359446	Δ1 bp	coding (831/1011 nt)	*PFLU1224 (wspF)*
WHS (2)	wrinkly	1359495	A→C	T294P (ACC→CCC)	*PFLU1224 (wspF)*
WHS (3)	wrinkly	1359538	C→T	P308L (CCC→CTC)	*PFLU1224 (wspF)*
WHS	wrinkly	1359552	(GCG)_4→3_	coding (937–939/1011 nt)	*PFLU1224 (wspF)*
WHS	wrinkly	1359554	(GCG)_4→5_	coding (939/1011 nt)	*PFLU1224 (wspF)*
ST/Lab (4)^b^	small	4830895	(GCAGGC)_2→1_	coding (8–13/1323 nt)	*PFLU4380 (dsbS)*
ST	small	4834252	Δ110 bp	fusion of 2 predicted ORFs	*[PFLU4383 (dsbE)]-[PFLU4384 (dsbG)]*
Lab^b^	small	4831698	A→C	Intergenic (114/49)	*PFLU4381 (dsbR)←/*→*PFLU4382 (dsbD)*
ST	wrinkly	5707197	G→A	Q157* (CAA→TAA)	*PFLU5211 (awsX/YfiR)*
ST	wrinkly	5707256	A→C	I137S (ATC→AGC)	*PFLU5211 (awsX/YfiR)*
WHS^b^	wrinkly	5707317	Δ33bp	coding (317–349/573 nt)	*PFLU5211 (awsX/YfiR)*
WHS	wrinkly	5707334	G→T	A111E (GCG→GAG)	*PFLU5211 (awsX/YfiR)*
ST/WHS (5)^b^	wrinkly	5707411	Δ33 bp	coding (223–255/573 nt)	*PFLU5211 (awsX/YfiR)*
ST (2)	wrinkly	5857638	G→A	M992I (ATG→ATA)	*PFLU5329 (mwsR/morA)*
ST (3)	wrinkly	5857733	Δ9 bp	coding (3071–3079/3852 nt)	*PFLU5329 (mwsR/morA)*
A (3)^b^	wrinkly	5858369	C→T	A1236V (GCC→GTC)	*PFLU5329 (mwsR/morA)*
**Lab** ^b^	**fuzzy**	**541214**	**C**→**T**	Q11* (CAG→**TAG**)	***PFLU0478 (fuzY)***
Lab	fuzzy	541772	Δ13 bp	coding (589–601/1149 nt)	*PFLU0478 (fuzY)*

a Lab refers to our research lab; count refers to the number of parallel mutations that occurred in different populations. Bolded mutants were those used in phenotypic studies.

b Denotes presence of secondary mutations; see Table S1 for details.

## Data Availability

All sequencing data are available at NCBI under Bioproject PRJNA1284392.

## References

[1] Stewart PS, Franklin MJ. 2008. Physiological heterogeneity in biofilms. Nat Rev Microbiol 6:199–210. 10.1038/nrmicro1838 18264116

[2] Poltak SR, Cooper VS. 2011. Ecological succession in long-term experimentally evolved biofilms produces synergistic communities. ISME J 5:369–378. 10.1038/ismej.2010.136 20811470 PMC3105725

[3] Traverse CC, Mayo-Smith LM, Poltak SR, Cooper VS. 2013. Tangled bank of experimentally evolved *Burkholderia* biofilms reflects selection during chronic infections. Proc Natl Acad Sci USA 110:E250–E259. 10.1073/pnas.1207025110 23271804 PMC3549113

[4] Ellis CN, Traverse CC, Mayo-Smith L, Buskirk SW, Cooper VS. 2015. Character displacement and the evolution of niche complementarity in a model biofilm community. Evolution (N Y) 69:283–293. 10.1111/evo.12581 PMC4335599 25494960

[5] Nadell CD, Drescher K, Foster KR. 2016. Spatial structure, cooperation and competition in biofilms. Nat Rev Microbiol 14:589–600. 10.1038/nrmicro.2016.84 27452230

[6] Cooper VS, Warren TM, Matela AM, Handwork M, Scarponi S. 2019. EvolvingSTEM: a microbial evolution-in-action curriculum that enhances learning of evolutionary biology and biotechnology. Evo Edu Outreach 12:12. 10.1186/s12052-019-0103-4 PMC7328067 32647555

[7] Spiers AJ. 2014. Getting Wrinkly Spreaders to demonstrate evolution in schools. Trends Microbiol 22:301–303. 10.1016/j.tim.2014.03.007 24881493

[8] Taylor MB, Warwick AR, Skophammer R, Boyer JM, Geck RC, Gunkelman K, Walson M, Rowley PA, Dunham MJ. 2024. yEvo: a modular eukaryotic genetics and evolution research experience for high school students. Ecol Evol 14:e10811. 10.1002/ece3.10811 38192907 PMC10771926

[9] Kassen R, Rainey PB. 2004. The ecology and genetics of microbial diversity. Annu Rev Microbiol 58:207–231. 10.1146/annurev.micro.58.030603.123654 15487936

[10] Habets M, Rozen DE, Hoekstra RF, de Visser J. 2006. The effect of population structure on the adaptive radiation of microbial populations evolving in spatially structured environments. Ecol Lett 9:1041–1048. 10.1111/j.1461-0248.2006.00955.x 16925653

[11] Harris KB, Flynn KM, Cooper VS. 2021. Polygenic adaptation and clonal interference enable sustained diversity in experimental *Pseudomonas aeruginosa* populations. Mol Biol Evol 38:5359–5375. 10.1093/molbev/msab248 34410431 PMC8662654

[12] Røder HL, Sørensen SJ, Burmølle M. 2016. Studying bacterial multispecies biofilms: where to start? Trends Microbiol 24:503–513. 10.1016/j.tim.2016.02.019 27004827

[13] Trubenová B, Roizman D, Moter A, Rolff J, Regoes RR. 2022. Population genetics, biofilm recalcitrance, and antibiotic resistance evolution. Trends Microbiol 30:841–852. 10.1016/j.tim.2022.02.005 35337697

[14] Hall-Stoodley L, Costerton JW, Stoodley P. 2004. Bacterial biofilms: from the natural environment to infectious diseases. Nat Rev Microbiol 2:95–108. 10.1038/nrmicro821 15040259

[15] Flemming H-C, Wuertz S 2019. Bacteria and archaea on Earth and their abundance in biofilms. Nat Rev Microbiol 17:247–260. 10.1038/s41579-019-0158-9 30760902

[16] Pestrak MJ, Chaney SB, Eggleston HC, Dellos-Nolan S, Dixit S, Mathew-Steiner SS, Roy S, Parsek MR, Sen CK, Wozniak DJ. 2018. *Pseudomonas aeruginosa* rugose small-colony variants evade host clearance, are hyper-inflammatory, and persist in multiple host environments. PLoS Pathog 14:e1006842. 10.1371/journal.ppat.1006842 29394295 PMC5812653

[17] Cooper VS, Staples RK, Traverse CC, Ellis CN. 2014. Parallel evolution of small colony variants in *Burkholderia cenocepacia* biofilms. Genomics 104:447–452. 10.1016/j.ygeno.2014.09.007 25263109

[18] O’Rourke D, FitzGerald CE, Traverse CC, Cooper VS. 2015. There and back again: consequences of biofilm specialization under selection for dispersal. Front Genet 6:18. 10.3389/fgene.2015.00018 25717335 PMC4324302

[19] Flynn KM, Dowell G, Johnson TM, Koestler BJ, Waters CM, Cooper VS. 2016. Evolution of ecological diversity in biofilms of *Pseudomonas aeruginosa* by altered cyclic diguanylate signaling. J Bacteriol 198:2608–2618. 10.1128/JB.00048-16 27021563 PMC5019052

[20] Turner CB, Marshall CW, Cooper VS. 2018. Parallel genetic adaptation across environments differing in mode of growth or resource availability. Evol Lett 2:355–367. 10.1002/evl3.75 30283687 PMC6121802

[21] Santos-Lopez A, Marshall CW, Scribner MR, Snyder DJ, Cooper VS. 2019. Evolutionary pathways to antibiotic resistance are dependent upon environmental structure and bacterial lifestyle. eLife 8:e47612. 10.7554/eLife.47612 31516122 PMC6814407

[22] Scribner MR, Santos-Lopez A, Marshall CW, Deitrick C, Cooper VS. 2020. Parallel evolution of tobramycin resistance across species and environments. mBio 11:e00932–20. 10.1128/mBio.00932-20 32457248 PMC7251211

[23] Mhatre E, Snyder DJ, Sileo E, Turner CB, Buskirk SW, Fernandez NL, Neiditch MB, Waters CM, Cooper VS. 2020. One gene, multiple ecological strategies: a biofilm regulator is a capacitor for sustainable diversity. Proc Natl Acad Sci USA 117:21647–21657. 10.1073/pnas.2008540117 32817433 PMC7474642

[24] Henriksen N, Hansen MF, Kiesewalter HT, Russel J, Nesme J, Foster KR, Svensson B, Øregaard G, Herschend J, Burmølle M. 2022. Biofilm cultivation facilitates coexistence and adaptive evolution in an industrial bacterial community. npj Biofilms Microbiomes 8:1–8. 10.1038/s41522-022-00323-x 35858930 PMC9300721

[25] Oakley JL, Weiser R, Powell LC, Forton J, Mahenthiralingam E, Rye PD, Hill KE, Thomas DW, Pritchard MF. 2021. Phenotypic and genotypic adaptations in Pseudomonas aeruginosa biofilms following long-term exposure to an alginate oligomer therapy. mSphere 6:e01216–20. 10.1128/mSphere.01216-20 33472983 PMC7845618

[26] Trampari E, Holden ER, Wickham GJ, Ravi A, Martins L de O, Savva GM, Webber MA. 2021. Exposure of Salmonella biofilms to antibiotic concentrations rapidly selects resistance with collateral tradeoffs. npj Biofilms Microbiomes 7:1–13. 10.1038/s41522-020-00178-0 33431848 PMC7801651

[27] Koza A, Kusmierska A, McLaughlin K, Moshynets O, Spiers AJ. 2017. Adaptive radiation of *Pseudomonas fluorescens* SBW25 in experimental microcosms provides an understanding of the evolutionary ecology and molecular biology of A-L interface biofilm formation. FEMS Microbiol Lett 364:fnx109. 10.1093/femsle/fnx109 28535292

[28] Rainey PB, Travisano M. 1998. Adaptive radiation in a heterogeneous environment. Nature 394:69–72. 10.1038/27900 9665128

[29] McDonald MJ, Gehrig SM, Meintjes PL, Zhang X-X, Rainey PB. 2009. Adaptive divergence in experimental populations of *Pseudomonas fluorescens*. IV. Genetic constraints guide evolutionary trajectories in a parallel adaptive radiation. Genetics 183:1041–1053. 10.1534/genetics.109.107110 19704015 PMC2778958

[30] Jenal U, Reinders A, Lori C. 2017. Cyclic di-GMP: second messenger extraordinaire. Nat Rev Microbiol 15:271–284. 10.1038/nrmicro.2016.190 28163311

[31] Laventie B-J, Jenal U 2020. Surface sensing and adaptation in bacteria. Annu Rev Microbiol 74:735–760. 10.1146/annurev-micro-012120-063427 32905753

[32] Malone JG, Williams R, Christen M, Jenal U, Spiers AJ, Rainey PB. 2007. The structure–function relationship of WspR, a *Pseudomonas fluorescens* response regulator with a GGDEF output domain. Microbiology (Reading) 153:980–994. 10.1099/mic.0.2006/002824-0 17379708

[33] Spiers AJ, Kahn SG, Bohannon J, Travisano M, Rainey PB. 2002. Adaptive divergence in experimental populations of *Pseudomonas fluorescens*. I. Genetic and phenotypic bases of wrinkly spreader fitness. Genetics 161:33–46. 10.1093/genetics/161.1.33 12019221 PMC1462107

[34] Spiers AJ, Bohannon J, Gehrig SM, Rainey PB. 2003. Biofilm formation at the air–liquid interface by the *Pseudomonas fluorescens* SBW25 wrinkly spreader requires an acetylated form of cellulose. Mol Microbiol 50:15–27. 10.1046/j.1365-2958.2003.03670.x 14507360

[35] Ferguson GC, Bertels F, Rainey PB. 2013. Adaptive divergence in experimental populations of *Pseudomonas fluorescens*. V. Insight into the niche specialist fuzzy spreader compels revision of the model *Pseudomonas* radiation. Genetics 195:1319–1335. 10.1534/genetics.113.154948 24077305 PMC3832276

[36] Cai Y-M, Yu K-W, Liu J-H, Cai Z, Zhou Z-H, Liu Y, Wang T-F, Yang L. 2022. The c-di-GMP phosphodiesterase PipA (PA0285) regulates autoaggregation and Pf4 bacteriophage production in Pseudomonas aeruginosa PAO1. Appl Environ Microbiol 88:e0003922. 10.1128/aem.00039-22 35638845 PMC9238385

[37] Eilers K, Kuok Hoong Yam J, Morton R, Mei Hui Yong A, Brizuela J, Hadjicharalambous C, Liu X, Givskov M, Rice SA, Filloux A. 2022. Phenotypic and integrated analysis of a comprehensive *Pseudomonas aeruginosa* PAO1 library of mutants lacking cyclic-di-GMP-related genes. Front Microbiol 13:949597. 10.3389/fmicb.2022.949597 35935233 PMC9355167

[38] Eilers K, Hoong Yam JK, Liu X, Goh YF, To K-N, Paracuellos P, Morton R, Brizuela J, Hui Yong AM, Givskov M, Freibert S-A, Bange G, Rice SA, Steinchen W, Filloux A. 2024. The dual GGDEF/EAL domain enzyme PA0285 is a *Pseudomonas* species housekeeping phosphodiesterase regulating early attachment and biofilm architecture. J Biol Chem 300:105659. 10.1016/j.jbc.2024.105659 38237678 PMC10874727

[39] Wei Q, Leclercq S, Bhasme P, Xu A, Zhu B, Zhang Y, Zhang M, Wang S, Ma LZ. 2019. Diguanylate cyclases and phosphodiesterases required for basal-level c-di-GMP in *Pseudomonas aeruginosa* as revealed by systematic phylogenetic and transcriptomic analyses. Appl Environ Microbiol 85:e01194–19. 10.1128/AEM.01194-19 31444209 PMC6803301

[40] Brisson D 2018. Negative frequency-dependent selection is frequently confounding. Front Ecol Evol 6:10. 10.3389/fevo.2018.00010 34395455 PMC8360343

[41] States NL. 2013. Next generation science standards: for states, by states. National Academies Press. 25927122

[42] Schweizer HP. 2001. Triclosan: a widely used biocide and its link to antibiotics. FEMS Microbiol Lett 202:1–7. 10.1111/j.1574-6968.2001.tb10772.x 11506900

[43] McFarland AG, Bertucci HK, Littman E, Shen J, Huttenhower C, Hartmann EM. 2021. Triclosan tolerance is driven by a conserved mechanism in diverse *Pseudomonas* species. Appl Environ Microbiol 87:e02924–20. 10.1128/AEM.02924-20 33483311 PMC8091609

[44] Lind PA, Libby E, Herzog J, Rainey PB. 2019. Predicting mutational routes to new adaptive phenotypes. eLife 8:e38822. 10.7554/eLife.38822 30616716 PMC6324874

[45] O’Toole GA, Kolter R. 1998. Initiation of biofilm formation in *Pseudomonas fluorescens* WCS365 proceeds via multiple, convergent signalling pathways: a genetic analysis. Mol Microbiol 28:449–461. 10.1046/j.1365-2958.1998.00797.x 9632250

[46] Caplat P, Anand M, Bauch C. 2008. Symmetric competition causes population oscillations in an individual-based model of forest dynamics. Ecol Modell 211:491–500. 10.1016/j.ecolmodel.2007.10.002

[47] Chesson P 2000. Mechanisms of maintenance of species diversity. Annu Rev Ecol Syst 31:343–366. 10.1146/annurev.ecolsys.31.1.343

[48] Bantinaki E, Kassen R, Knight CG, Robinson Z, Spiers AJ, Rainey PB. 2007. Adaptive divergence in experimental populations of *Pseudomonas fluorescens*. III. Mutational origins of wrinkly spreader diversity. Genetics 176:441–453. 10.1534/genetics.106.069906 17339222 PMC1893022

[49] Xu A, Wang D, Wang Y, Zhang L, Xie Z, Cui Y, Bhamse P, Yu H, Zhang X-X, Li D, Ma LZ. 2022. Mutations in surface-sensing receptor WspA lock the Wsp signal transduction system into a constitutively active state. Environ Microbiol 24:1150–1165. 10.1111/1462-2920.15763 34499799

[50] O’Connor JR, Kuwada NJ, Huangyutitham V, Wiggins PA, Harwood CS. 2012. Surface sensing and lateral subcellular localization of WspA, the receptor in a chemosensory-like system leading to c-di-GMP production. Mol Microbiol 86:720–729. 10.1111/mmi.12013 22957788 PMC3501340

[51] Kessler C, Mhatre E, Cooper V, Kim W. 2021. Evolutionary divergence of the Wsp signal transduction systems in beta- and gammaproteobacteria. Appl Environ Microbiol 87:e0130621. 10.1128/AEM.01306-21 PMC8552884 34495711

[52] Kessler C, Kim W. 2022. Identification of Cyclic-di-GMP-modulating protein residues by bidirectionally evolving a social behavior in Pseudomonas fluorescens. mSystems 7:e0073722. 10.1128/msystems.00737-22 PMC9600634 36190139

[53] O’Neal L, Baraquet C, Suo Z, Dreifus JE, Peng Y, Raivio TL, Wozniak DJ, Harwood CS, Parsek MR. 2022. The Wsp system of *Pseudomonas aeruginosa* links surface sensing and cell envelope stress. Proc Natl Acad Sci USA 119:e2117633119. 10.1073/pnas.2117633119 PMC9170161 35476526

[54] Malone JG, Jaeger T, Spangler C, Ritz D, Spang A, Arrieumerlou C, Kaever V, Landmann R, Jenal U. 2010. YfiBNR mediates cyclic di-GMP dependent small colony variant formation and persistence in *Pseudomonas aeruginosa*. PLoS Pathog 6:e1000804. 10.1371/journal.ppat.1000804 PMC2837407 20300602

[55] Malone JG, Jaeger T, Manfredi P, Dötsch A, Blanka A, Bos R, Cornelis GR, Häussler S, Jenal U. 2012. The YfiBNR signal transduction mechanism reveals novel targets for the evolution of persistent *Pseudomonas aeruginosa* in cystic fibrosis airways. PLoS Pathog 8:e1002760. 10.1371/journal.ppat.1002760 PMC3375315 22719254

[56] Li S, Li T, Teng X, Lou X, Xu Y, Zhang Q, Bartlam M. 2018. Structural analysis of activating mutants of YfiB from *Pseudomonas aeruginosa* PAO1. Biochem Biophys Res Commun 506:997–1003. 10.1016/j.bbrc.2018.10.190 30404734

[57] Xu M, Yang X, Yang X-A, Zhou L, Liu T-Z, Fan Z, Jiang T. 2016. Structural insights into the regulatory mechanism of the *Pseudomonas aeruginosa* YfiBNR system. Protein Cell 7:403–416. 10.1007/s13238-016-0264-7 27113583 PMC4887326

[58] Yang X, Yang X-A, Xu M, Zhou L, Fan Z, Jiang T. 2015. Crystal structures of YfiR from *Pseudomonas aeruginosa* in two redox states. Biochem Biophys Res Commun 461:14–20. 10.1016/j.bbrc.2015.03.160 25849887

[59] Phippen CW, Mikolajek H, Schlaefli HG, Keevil CW, Webb JS, Tews I. 2014. Formation and dimerization of the phosphodiesterase active site of the *Pseudomonas aeruginosa* MorA, a bi-functional c-di-GMP regulator. FEBS Lett 588:4631–4636. 10.1016/j.febslet.2014.11.002 25447517

[60] Lam JS, Taylor VL, Islam ST, Hao Y, Kocíncová D. 2011. Genetic and functional diversity of Pseudomonas aeruginosa lipopolysaccharide. Front Microbio 2. 10.3389/fmicb.2011.00118 PMC3108286 21687428

[61] Lerouge I, Vanderleyden J. 2002. O-antigen structural variation: mechanisms and possible roles in animal/plant–microbe interactions. FEMS Microbiol Rev 26:17–47. 10.1111/j.1574-6976.2002.tb00597.x 12007641

[62] Lau PCY, Lindhout T, Beveridge TJ, Dutcher JR, Lam JS. 2009. Differential lipopolysaccharide core capping leads to quantitative and correlated modifications of mechanical and structural properties in *Pseudomonas aeruginosa* biofilms. J Bacteriol 191:6618–6631. 10.1128/JB.00698-09 19717596 PMC2795305

[63] Bardwell JCA, McGovern K, Beckwith J. 1991. Identification of a protein required for disulfide bond formation *in vivo*. Cell 67:581–589. 10.1016/0092-8674(91)90532-4 1934062

[64] Bardwell JC, Lee JO, Jander G, Martin N, Belin D, Beckwith J. 1993. A pathway for disulfide bond formation *in vivo*. Proc Natl Acad Sci USA 90:1038–1042. 10.1073/pnas.90.3.1038 8430071 PMC45806

[65] Bader MW, Xie T, Yu C-A, Bardwell JCA. 2000. Disulfide bonds are generated by quinone reduction. J Biol Chem 275:26082–26088. 10.1074/jbc.M003850200 10854438

[66] Berkmen M, Boyd D, Beckwith J. 2005. The nonconsecutive disulfide bond of Escherichia coli phytase (AppA) renders it dependent on the protein-disulfide isomerase, DsbC. J Biol Chem 280:11387–11394. 10.1074/jbc.M411774200 15642731

[67] Hiniker A, Bardwell JCA. 2004. *In vivo* substrate specificity of periplasmic disulfide oxidoreductases. J Biol Chem 279:12967–12973. 10.1074/jbc.M311391200 14726535

[68] Manta B, Boyd D, Berkmen M. 2019. Disulfide bond formation in the periplasm of Escherichia coli. EcoSal Plus 8. 10.1128/ecosalplus.ESP-0012-2018 PMC11573287 30761987

[69] Yu L, Cao Q, Chen W, Yang N, Yang C-G, Ji Q, Wu M, Bae T, Lan L. 2022. A novel copper-sensing two-component system for inducing Dsb gene expression in bacteria. Sci Bull (Beijing) 67:198–212. 10.1016/j.scib.2021.03.003 36546013

[70] Ha D-G, Richman ME, O’Toole GA. 2014. Deletion mutant library for investigation of functional outputs of cyclic diguanylate metabolism in *Pseudomonas aeruginosa* PA14. Appl Environ Microbiol 80:3384–3393. 10.1128/AEM.00299-14 24657857 PMC4018857

[71] Levins R 1963. Theory of fitness in a heterogeneous environment. II. Developmental flexibility and niche selection. Am Nat 97:75–90. 10.1086/282258

[72] Martin M, Hölscher T, Dragoš A, Cooper VS, Kovács ÁT. 2016. Laboratory evolution of microbial interactions in bacterial biofilms. J Bacteriol 198:2564–2571. 10.1128/JB.01018-15 27044625 PMC5019067

[73] Gloag ES, Marshall CW, Snyder D, Lewin GR, Harris JS, Santos-Lopez A, Chaney SB, Whiteley M, Cooper VS, Wozniak DJ. 2019. Pseudomonas aeruginosa interstrain dynamics and selection of hyperbiofilm mutants during a chronic infection. mBio 10:e01698–19. 10.1128/mBio.01698-19 31409682 PMC6692513

[74] Marvig RL, Sommer LM, Molin S, Johansen HK. 2015. Convergent evolution and adaptation of *Pseudomonas aeruginosa* within patients with cystic fibrosis. Nat Genet 47:57–64. 10.1038/ng.3148 25401299

[75] Smith EE, Buckley DG, Wu Z, Saenphimmachak C, Hoffman LR, D’Argenio DA, Miller SI, Ramsey BW, Speert DP, Moskowitz SM, Burns JL, Kaul R, Olson MV. 2006. Genetic adaptation by *Pseudomonas aeruginosa* to the airways of cystic fibrosis patients. Proc Natl Acad Sci USA 103:8487–8492. 10.1073/pnas.0602138103 16687478 PMC1482519

[76] Baym M, Kryazhimskiy S, Lieberman TD, Chung H, Desai MM, Kishony R. 2015. Inexpensive multiplexed library preparation for megabase-sized genomes. PLoS ONE 10:e0128036. 10.1371/journal.pone.0128036 26000737 PMC4441430

[77] Bolger AM, Lohse M, Usadel B. 2014. Trimmomatic: a flexible trimmer for Illumina sequence data. Bioinformatics 30:2114–2120. 10.1093/bioinformatics/btu170 24695404 PMC4103590

[78] Deatherage DE, Barrick JE. 2014. Identification of mutations in laboratory-evolved microbes from next-generation sequencing data using breseq. Methods Mol Biol 1151:165–188. 10.1007/978-1-4939-0554-6_12 24838886 PMC4239701

[79] FillouxA, RamosJL. 2014. Pseudomonas methods and protocols. Springer, New York, NY. https://link.springer.com/10.1007/978-1-4939-0473-0.

[80] Schindelin J, Arganda-Carreras I, Frise E, Kaynig V, Longair M, Pietzsch T, Preibisch S, Rueden C, Saalfeld S, Schmid B, Tinevez J-Y, White DJ, Hartenstein V, Eliceiri K, Tomancak P, Cardona A. 2012. Fiji: an open-source platform for biological-image analysis. Nat Methods 9:676–682. 10.1038/nmeth.2019 22743772 PMC3855844

[81] Rybtke MT, Borlee BR, Murakami K, Irie Y, Hentzer M, Nielsen TE, Givskov M, Parsek MR, Tolker-Nielsen T. 2012. Fluorescence-based reporter for gauging cyclic di-GMP levels in *Pseudomonas aeruginosa*. Appl Environ Microbiol 78:5060–5069. 10.1128/AEM.00414-12 22582064 PMC3416407

[82] Hartmann R, Jeckel H, Jelli E, Singh PK, Vaidya S, Bayer M, Rode DKH, Vidakovic L, Díaz-Pascual F, Fong JCN, Dragoš A, Lamprecht O, Thöming JG, Netter N, Häussler S, Nadell CD, Sourjik V, Kovács ÁT, Yildiz FH, Drescher K. 2021. Quantitative image analysis of microbial communities with BiofilmQ. Nat Microbiol 6:151–156. 10.1038/s41564-020-00817-4 33398098 PMC7840502

[83] Zhang X-X, Rainey PB. 2007. Construction and validation of a neutrally-marked strain of *Pseudomonas fluorescens* SBW25. J Microbiol Methods 71:78–81. 10.1016/j.mimet.2007.07.001 17669526

