# Supplementary material for: Genetic diversification of Pseudomonas fluorescens maintained by multi-niche selection within biofilms
Source: Appl Environ Microbiol. Author manuscript; Available in PMC 2026 Apr 30. (PMC13101516; doi:10.1128/aem.02499-25)

SUPPLEMENTARY MATERIAL

Genetic diversification of *Pseudomonas fluorescens* maintained by multi-niche selection within biofilms

Abigail M. Matela[12], Colton W. Siatkowski[12], Changhua Yan[12], Sachin Thiagarajan[1], Erin M. Nawrocki, Vaughn S. Cooper[12]

Table S1. Complete details of mutant genotypes, including secondary mutations (XLS).

Table S2. Details of mutations detected during the evolution experiment using population-wide whole-genome sequencing (XLS)

Table S3. Linear regressions of frequencies of *bmo* (PFLU0185) between the ancestor (Afreq), *wsp* mutant (Wfreq), and *fzy* mutant (Ffreq), as shown in Figure 8. The significant negative slopes indicate competition.

Model Equation	Std. Error	t value	R-squared	p-value
Pfreq = (-0.99)Afreq + 0.91	0.07	-14.69	0.79	<0.0001
Pfreq = (-0.7)Wfreq + 0.46	0.32	-2.22	0.08	<0.05
Pfreq = (-1.84)Ffreq + 0.48	0.58	-3.14	0.15	<0.001

Table S4. Plasmids used in this study

Name	Plasmid	Source/Reference
pTNS3	Helper plasmid for site-specific Tn7 transposition	Choi et al. (Choi et al., 2008)
pBT270	pUC18-mini-Tn7T2-PA1/04/03-Gm-GFP	Zhao et al. (Zhao et al., 2013)
pBT277	pUC18-mini-Tn7T2-PA1/04/03-Gm-mCherry	Zhao et al. (Zhao et al., 2013)

Supplementary Curriculum can be downloaded at http://evolvingstem.org/curriculum

Fig. S1. *Pseudomonas fluorescens* lacks twitching motility. Twitching motility was measured on LB-Lennox Agar Plates after 24 hours of growth. PAO1 (A) exhibits a twitching phenotype, indicated by the halo surrounding the dark red colony in the center of the plate where inoculation occurred. *P. fluorescens* ancestor (B), *pflu0185* mutants (C), *fuzY* mutants (D), and *wspF* mutants (E) show no signs of twitching motility as all four genotypes lack a halo surrounding the inoculation point.

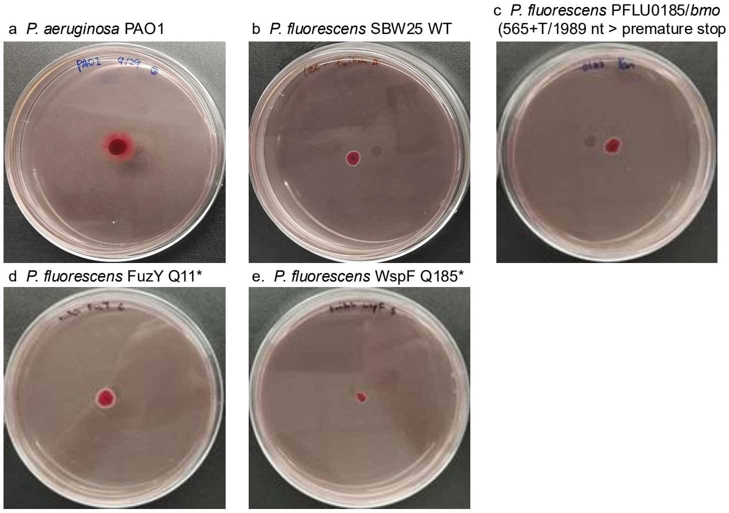

Fig. S2. Planktonic growth is inhibited by triclosan.

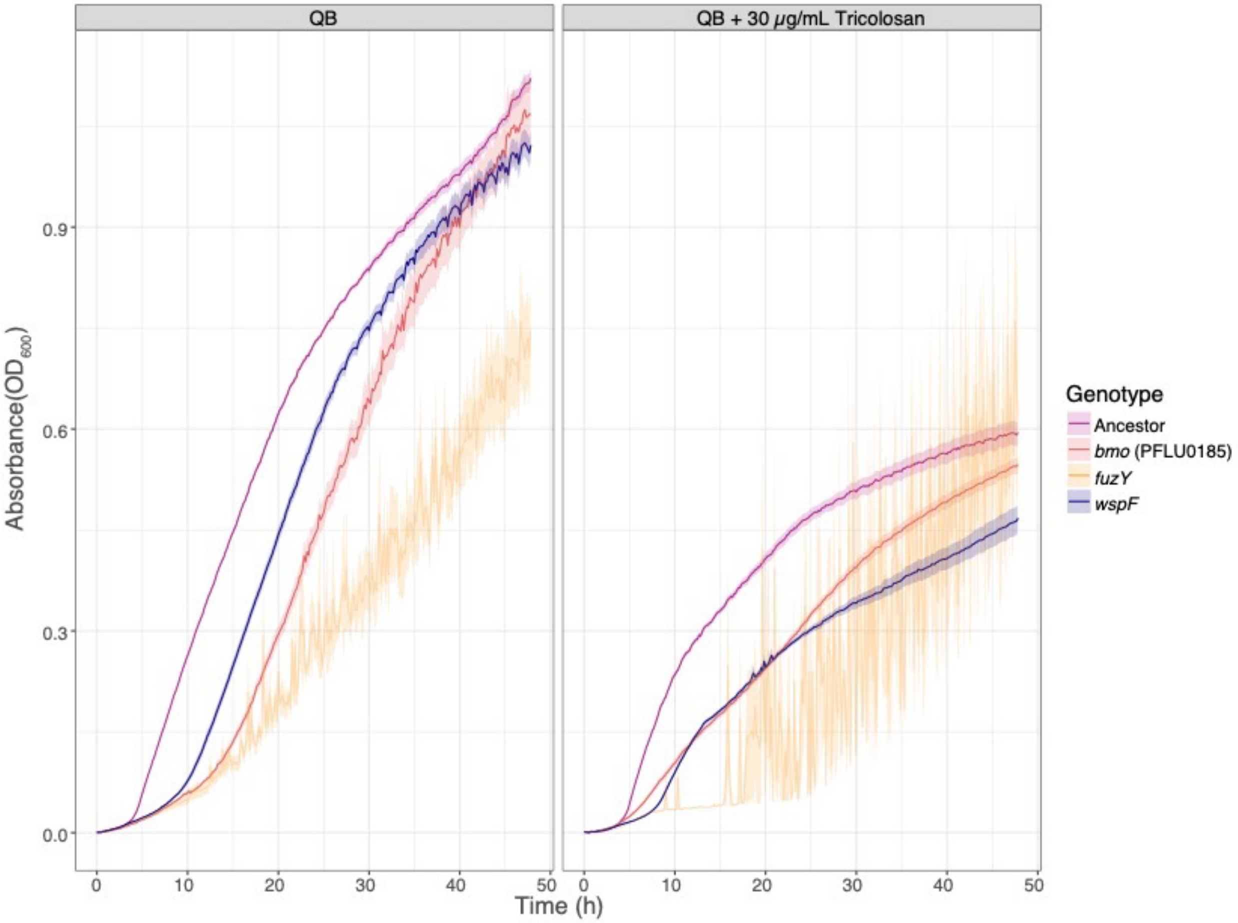

Fig. S3. Confocal microscopy of representative *P. fluorescens* mutants mixed in pairs shows differentiated biofilm phenotypes. Methods replicate those of Fig 6. Text color of labels indicates fluorescent marker in that genotype; red = mCherry, green = eGFP.

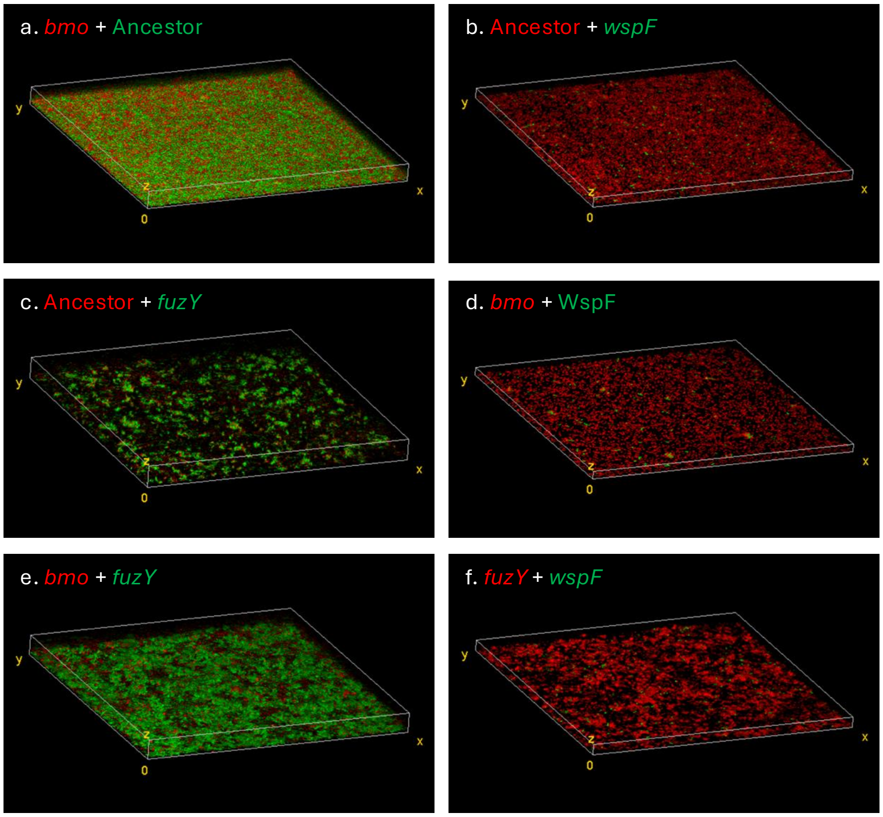

Fig. S4. Colony morphology of small colony variants with mutations in *dsb* genes and their differential uptake of Congo Red dye.

PFLU4380 & PFLU4383-dsbG (PFLU4384)

4 SCVs from evolved bead populations

1. SciTech 2019 10: (GCAGGC)$_{2\rightarrow1}$ coding (8-13)
2. SciTech 2019 16: (GCAGGC)$_{2\rightarrow1}$ coding (8-13)
3. SciTech 2019 19: (GCAGGC)$_{2\rightarrow1}$ coding (8-13)
4. SciTech 2019 20: **Δ**110bp

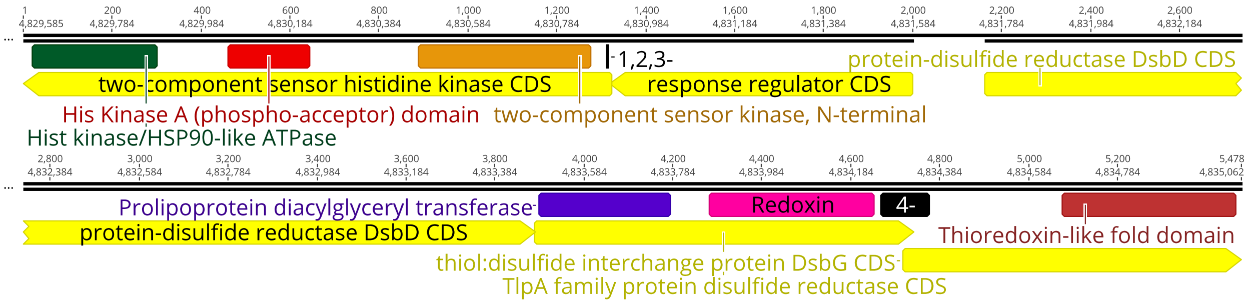

Spot (A) and colony (B) phenotypes of the ancestor (circled) in comparison to SciTech20 mutant on ½ tsoy indicator agar.

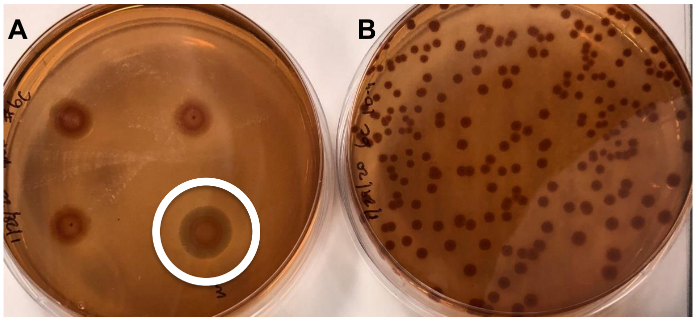

Supplement: Supp Tables and Figs — Supplemental material (AEM02499-25-s0001.pdf). Tables S3 and S4; Fig. S1 to S4. [file NIHMS2165571-supplement-Supp_Tables_and_Figs.pdf]
